# Three-dimensional organization of pyrrolo[3,2-b]pyrrole-based triazine framework using nanostructural spherical carbon: enhancing electrochemical performance of materials for supercapacitors

**DOI:** 10.1038/s41598-023-37708-7

**Published:** 2023-07-03

**Authors:** Agnieszka Hryniewicka, Joanna Breczko, Gabriela Siemiaszko, Anthony N. Papathanassiou, Kinga Góra-Marek, Karolina A. Tarach, Krzysztof Brzezinski, Anna Ilnicka, Artur P. Terzyk, Karolina H. Markiewicz, Luis Echegoyen, Marta E. Plonska-Brzezinska

**Affiliations:** 1 Department of Organic Chemistry, Faculty of Pharmacy with the Division of Laboratory Medicine, Medical University of Bialystok, Mickiewicza 2A, 15-222 Bialystok, Poland; 2 Faculty of Chemistry, University of Bialystok, Ciolkowskiego 1K, 15-245 Bialystok, Poland; 3 Physics Department, Condensed Matter Physics Section, National and Kapodistrian University of Athens, Panepistimiopolis, 15784 Zografos, Athens, Greece; 4 Faculty of Chemistry, Jagiellonian University in Krakow, Gronostajowa 2, 30-387 Krakow, Poland; 5 Department of Structural Biology of Prokaryotic Organisms, Institute of Bioorganic Chemistry, Polish Academy of Sciences, Noskowskiego 12/14, 61-074 Poznan, Poland; 6 Faculty of Chemistry, Nicolaus Copernicus University in Torun, Gagarin 7, 87-100 Torun, Poland; 7 Department of Chemistry, University of Texas at El Paso, 500 W. University Ave., El Paso, TX 79968 USA

**Keywords:** Electrochemistry, Materials chemistry

## Abstract

Covalent triazine-based frameworks have attracted much interest recently due to their high surface area and excellent thermal and electrochemical stabilities. This study shows that covalently immobilizing triazine-based structures on spherical carbon nanostructures results in the organization of micro- and mesopores in a three-dimensional manner. We selected the nitrile-functionalized pyrrolo[3,2-b]pyrrole unit to form triazine rings to construct a covalent organic framework. Combining spherical carbon nanostructures with the triazine framework produced a material with unique physicochemical properties, exhibiting the highest specific capacitance value of 638 F g^−1^ in aqueous acidic solutions. This phenomenon is attributed to many factors. The material exhibits a large surface area, a high content of micropores, a high content of graphitic N, and N-sites with basicity and semi-crystalline character. Thanks to the high structural organization and reproducibility, and remarkably high specific capacitance, these systems are promising materials for use in electrochemistry. For the first time, hybrid systems containing triazine-based frameworks and carbon nano-onions were used as electrodes for supercapacitors.

## Introduction

Although the techniques for functionalizing organic and inorganic molecules are well established, there are still many problems in controlling the structures of materials that are made with relatively large molecules. Porous materials are currently at the center of attention in various fields of science. The porous materials that are well-designed may serve as the basis for electrocatalysts, electrochemical capacitors, microscale supercapacitors, advanced photovoltaic devices, and microscale sensors, all of which exhibit significant advantages in performance over the current state-of-the-art technologies^1–3^. So far, carbon materials are commonly used in electrochemistry, particularly as electrode materials^4^. Although these materials have many advantages, it is still looking for materials with better parameters that allow use as commercial supercapacitors (SCs). SCs should be characterized by a high power density, fast charging/discharging, and long-term mechanical and electrochemical stability^5^. The mechanism for energy storage in SCs is frequently based on the separation of charges at the carbon electrode/electrolyte interface^6^. The combination of the materials with differential chemical characters increases the capacity and porosity of electrodes, reaching better electrochemical parameters^7,8^. Moreover, combining faradaic and non-faradaic processes in hybrid capacitors can achieve higher energy and power density while maintaining long-term electrochemical stability^9^.

Covalent triazine-based frameworks (CTFs) have attracted much interest in recent years due to their high surface area as well as excellent thermal and electrochemical stabilities^10^. Their synthesis relies on forming a triazine ring as a covalent bond between organic building blocks to achieve an extended porous framework^11^. Due to the stable triazine-based linkage, high intrinsic nitrogen content, and ability to add various heteroatoms to their structures, CTFs have the potential to be utilized in several applications, such as photo- and electrocatalysis, batteries, gas adsorption, separation, and pollutant removal^12^. The main advantage of CTFs is that a regular two-dimensional (2D) organization is obtained without using auxiliary structure-directing agents^10^. The pore size can be adjusted by simply tuning the size and structural features of the starting monomer. However, the organization of triazine framework sheets via π-stacking has significant limitations to obtaining regular organization in three-dimensional (3D) architectures^13–15^.

Hybrid materials contain two or more constituents and are emerging as a very promising class of materials due to the diverse, complementary nature of the properties inherent of these different classes of materials^12^. The diversity of resultant properties and materials used in the construction of hybrids leads to a very broad range of application areas generated by engaging very different research communities^16^. There are only a few reports focusing on CTF and carbon materials. The carboxylated multi-walled carbon nanotubes (MWCNTs) and CTFs were used for simultaneous electrochemical detection of hydroquinone and catechol^13^ and as a multi-functional separator^17^. Lamont et al. proposed functionalization of CTFs by an ethereal linkage between hydroxyl groups decorating the surface of MWCNTs and CTFs, which were applied for selective electrochemical conversion of CO_2_–CO^14^. MWCNT-CTF was used as a versatile sulfur host and find application in rechargeable batteries^18^. Graphene oxide (GO) was also used as a component of CTF hybrids. Liu et al.^15^ synthesized a composite containing CTF and GO for boosting photocatalytic H_2_ evolution. Shen et al.^19^ synthesized a series of CTF-GO aerogels to remove traces of benzophenone derivatives from water, and Yuan et al.^20^ used CTF and reduced form of GO for an emerging high-performance cathode. The improved electrochemical, photo- and electrocatalytic properties for the hybrid materials compared to pristine CTFs provides evidence of the effectiveness of rational structure engineering. Although the abovementioned reports indicate some improvement in the physicochemical properties of the hybrid materials, they do not discuss the correlation between the structure of the synthesized materials and the influence of specific parameters on their applications. Moreover, only in one abovementioned report, CTF is covalently immobilized on carbon nanostructure^14^. In this study, we covalently immobilized triazine-based structures on spherical carbon nanostructures, called carbon nano-onions (CNOs), which results in the organization of micro- and mesopores in a 3D manner.

Among other features of CNO, which will be of crucial importance for the electrochemical properties of nanocomposites, the following should be emphasized: a high conductivity, high thermal stability, high specific surface area (SSA) and high chemical reactivity in comparison to other carbon nanostructures. Briefly, CNOs formed at high temperatures (ca. 1800 °C) from ND particles show the best performance in terms of conductivity^21^. Our studies showed that electron conduction through the junctions occurs by a superexchange mechanism, and the conductivity of the CNOs was ~ 71.8 µS, similar to a metallic behavior. Thermogravimetric analyses of the CNOs showed their high thermal stability in an air atmosphere, even higher than that of C_60_^22^. CNOs obtained from annealed NDs at the temperature range between 1300 and 1800 °C have also displayed high SSA values. The SSA determined by N_2_ gas adsorption was between 380 and 600 m^2^ g^−1^^23^, lower than that of many carbon materials, such as activated carbon. Still, it is fully accessible for ion adsorption in electrochemical devices^24^. A comparison between different energy storage devices designed for microelectronics power applications, tested under the same dynamic conditions, clearly showed that using the spherical nanostructures of CNOs resulted in high power and high energy delivery. Due to their high curvature, the reactivity of CNOs is very high**,** comparable to that of other carbon nanostructures^25–28^. Combining the advantages of CTF with the unique properties of spherical CNOs, 3D CTF-CNO nanocomposites will be synthesized whose physicochemical properties are up-and-coming in electrochemistry. These hybrid systems have never been used as electrodes for SCs, where the porous characteristic is crucial for their applications.

## Results and discussion

### Synthesis of 2CNPP-based CTFs

**Figure 1:**
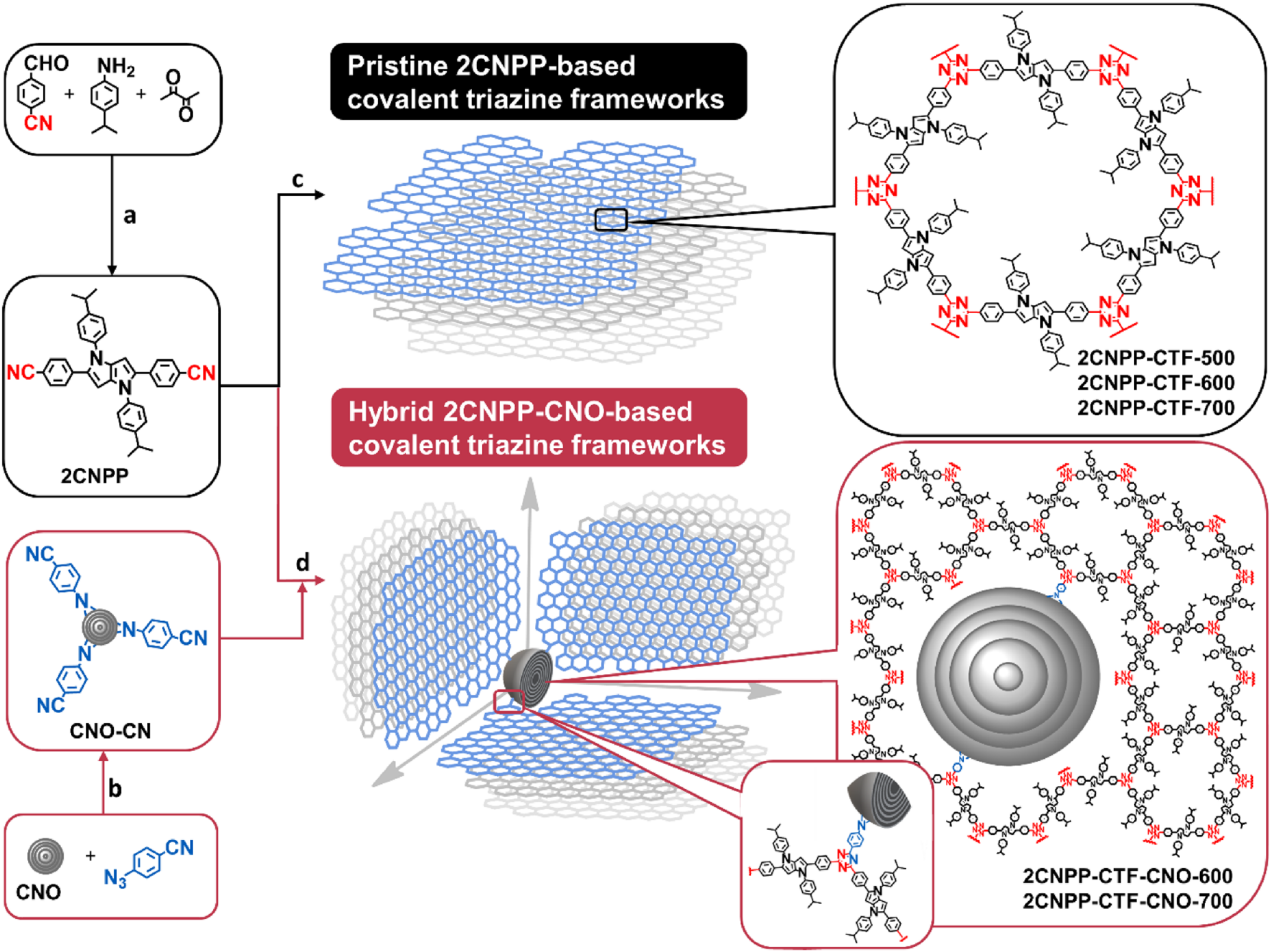
Representation of the synthetic pathways for CTFs and CTF-CNOs and of the organization of CTFs on CNO surfaces in 3D. Reagents and reaction conditions: (**a**) Fe(ClO_4_)_3_·H_2_O, air, AcOH/toluene, 50 °C, 12 h; (**b**) dichlorobenzene, 120 °C, 12 h; (**c**) ZnCl_2_ (20 eq), 500, 600 or 700 °C, 48 h; (**d**) ZnCl_2_, 600 or 700 °C, 48 h.

Our strategy involves the use of nanostructured carbon, CNOs, to be explored as spherical conductive platforms for the organization of pores in a 3D manner using regular triazine-based structures. The main reasons for using CNOs were to create porous carbonaceous materials and to modify the 3D architecture and organize the porous structure in a highly reproducible manner, which would ensure that an orderly distribution of pores with a defined size would be obtained in the final materials. Tetraarylpyrrolo[3,2-b]pyrrole, symmetrically substituted with cyanobenzene units, was selected to construct a CTF with a strongly flattened core and pores with a desired diameter (Fig. 1). This is the first report in which the pyrrolo[3,2-b]pyrrole unit constitutes a linker between triazine rings in a CTF. The organization of a typical CTF in a 2D manner corresponds to a graphene-like single layer. The surface of the CNOs is rich in *sp*^*2*^-hybridized carbon atoms, which causes the triazine frameworks to grow in a multidirectional manner on the spherical surface of the CNOs to form a 3D organized porous structure (Fig. 1). We designed 1,4-bis(4-isopropylphenyl)-2,5-bis(4-cyano-phenyl)-1,4-dihydropyrrolo[3,2-b]pyrrole (**2CNPP**) as the ideal monomer to construct a CTF due to the linear and planar structure of the molecule, the symmetrical substitution with the –CN group, and the presence of a strongly flattened core^10^. In addition to two cyanoaryl substituents in the monomer, isopropyl benzene was chosen as a substituent to nitrogen atoms, which may cause spatial hindrance in the resulting CTF.

The synthesis of **2CNPP** has never been described. **2CNPP** was synthesized by a condensation reaction catalyzed by iron(III) perchloride hydrate^29^. The **2CNPP-CTFs** were synthesized by an ionothermal process that was catalyzed by excess of ZnCl_2_. Three different pyrolysis temperatures (500, 600, or 700 °C) were used to change the surface composition of these materials^30^. It was previously observed that the porosity of pristine CTF increased with an increase in ZnCl_2_ concentration; however, the structure of the CTF became more amorphous as the catalyst concentration was increased^10^. To achieve complete dissolution of the monomers it was necessary to use a significant excess of ZnCl_2_ (approximately 20 molar equivalents). Melted ZnCl_2_ acts as a Lewis-acid catalyst, solvent and porogen for polymerization.

To ensure the CTFs and CNOs were in contact, the CNO structure was covalently functionalized utilizing the reaction with 4-azidobenzonitryl to form an aziridine ring with the surface of CNOs (**CNO-CN**) (Fig. 1)^28^. The **2CNPP-CTF-CNOs** were prepared analogously to the **2CNPP-CTFs**. The difference was the addition of **CNO-CNs** (5 wt%) to the monomer (**2CNPP**) and ZnCl_2_ before being subjected to pyrolysis at 600 or 700 °C. All CTFs were finally obtained as black monolithic materials with high yields (88–97%), what suggest low amounts of side reactions during these trimerization processes. The synthesis yield slightly decreases with increasing pyrolysis temperature.

The thermal stability of the synthesized materials was confirmed using thermogravimetric analysis (TGA) (Supplementary Fig. 12). The **2CNPP-CTF** and **2CNPP-CTF-CNO** materials decompose almost totally in an air atmosphere, showing one significant weight loss between 450 and 680 °C with a maximum degradation rate in the range 550–640 °C (Supplementary Fig. 12). One degree of degradation indicates a high chemical and structural similarity of the obtained carbonaceous materials. Pyrolyzed materials show high thermal stability up to 450 °C, and their decomposition profiles are similar, degrading continuously over the entire temperature range applied. The lowest thermal stability was observed for the material obtained at 500 °C (**2CNPP-CTF-500**). Materials pyrolyzed at higher temperatures have a higher degree of structural organization and graphitization, resulting in higher thermal stability.

### FT-IR and Raman spectroscopy

**Figure 2:**
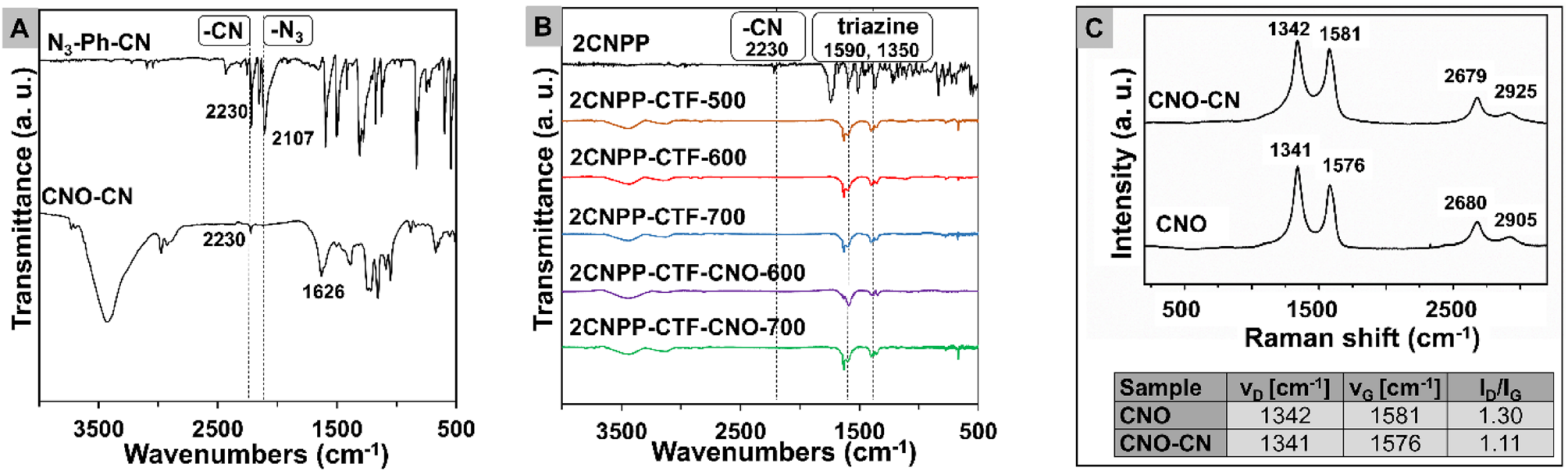
(**A**) FTIR spectra of 4-azidobenzonitrile and covalently modified CNO (**CNO-CN**); (**B**) FTIR spectra of the **2CNPP-CTF** and **2CNPP-CTF-CNO** materials; (**C**) Raman spectra of pristine and covalently modified CNO (**CNO-CN**) and D and G bands obtained at a laser excitation energy of 514 nm and the relative *I*_*D*_*/I*_*G*_.

The Fourier transform infrared (FT-IR) spectrum of **CNO-CN** reveals the presence of nitrile groups on the surface of CNOs (Fig. 2A). The absorption band at 2230 cm^−1^ can be attributed to the stretching vibrations of the –C=N group. The spectral feature at 1626 cm^−1^ corresponds to the aziridine ring (by which the organic molecule is immobilized on the spherical carbon material)^28^. Moreover, in comparison to the organic substrate (4-azidobenzonitryl), the absence of a band at 2107 cm^−1^ (characteristic of the stretching vibration of the –N_3_ group in organic azide) in the **CNO-CN** spectrum indicates that all azide groups reacted with the CNO surface to form aziridine bonds (–N < CNO). The trimerization of **2CNPP** was confirmed by infrared spectroscopy (Fig. 2B). All **2CNPP-CTFs** exhibit bands at ca. 1590 and 1350 cm^−1^, which is characteristic of the stretching vibrations of the triazine unit. The complete trimerization of the monomer was established by the absence of a carbonitrile band at 2230 cm^−1^^31^.

The Raman spectroscopic studies support our findings that the CNOs are covalently modified with phenylnitrile groups (Fig. 2C). The Raman spectrum for CNOs shows four characteristic bands at approximately 1342 (D band), 1581 (G band), 2679 and 2925 cm^−1^. Two prominent bands, D and G, are attributed to the* A*_*1g*_ vibration mode of the disorder-induced Raman line and the *E*_*2g*_ vibration of *sp*^2^-hybridized carbon atoms in the ordered graphite structure, respectively^32^. Second-order features between 2600 and 3000 cm^−1^ are the overtones of fundamental tones due to symmetry breaking^33^.

The ratio of intensities between the D and G bands (*I*_*D*_/*I*_*G*_) depends on the carbon type, and it could confirm the covalent functionalization of the CNOs^34^. An increase of the G band intensity in the Raman spectrum and a decrease in the *I*_*D*_*/I*_*G*_ ratio between CNOs and **CNO-CNs** is observed (Fig. 2C). The functionalization of CNOs with an aziridine ring causes the rupture of the outer shell and, therefore, the change in the hybridization of C atoms to *sp*^3^. At the same time, the number of C atoms with *sp*^2^ hybridization increases, resulting from the substitution of the aromatic ring with the CN groups.

### Determination of elemental composition and crystallinity

**Figure 3:**
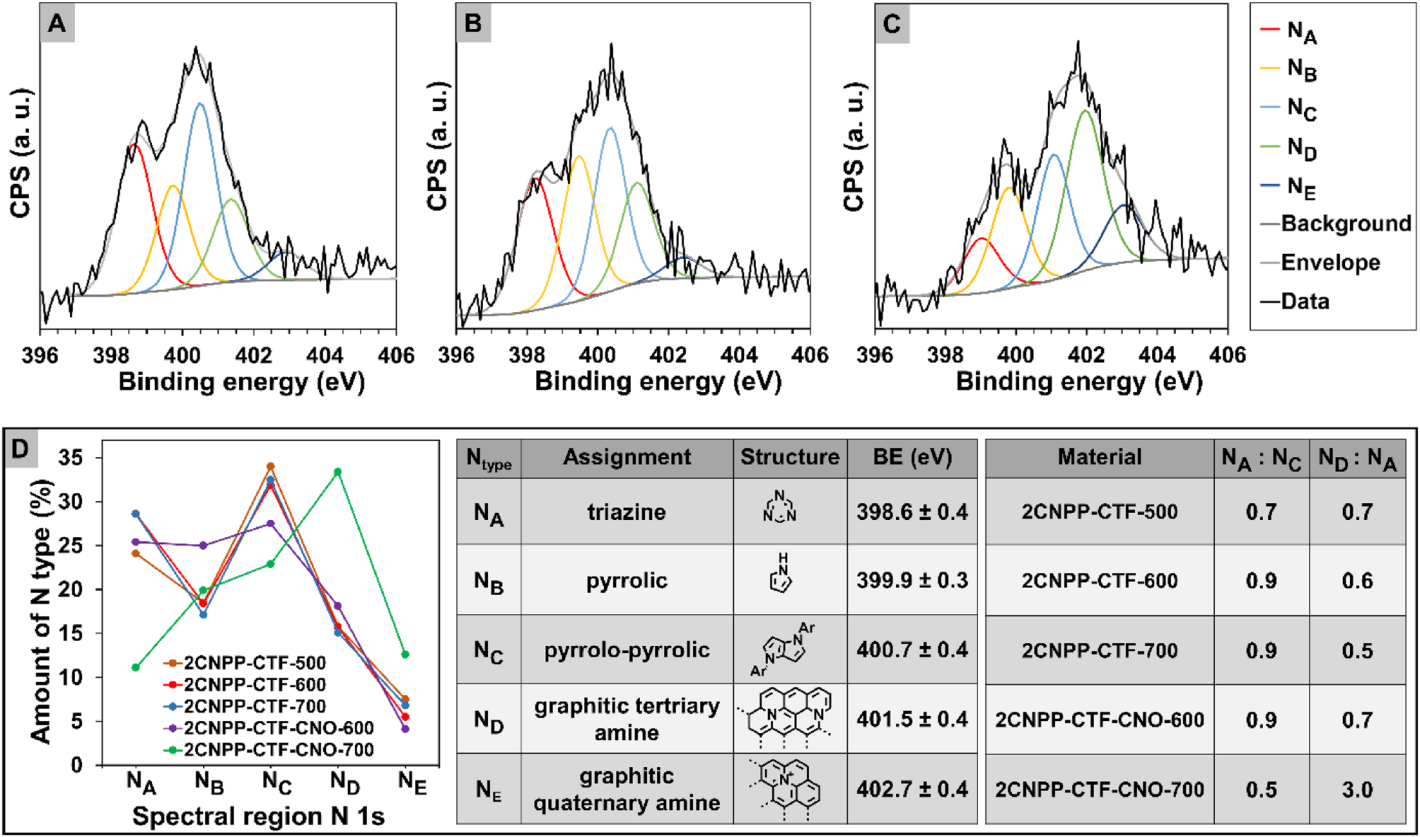
XPS spectra of the N 1*s* spectral region of (**A**) **2CNPP-CTF-600**, (**B**) **2CNPP-CTF-CNO-600** and (**C**) **2CNPP-CTF-CNO-700**. (**D**) Comparison of N distribution in all CTFs, description of the different types of N, and comparison of the particular N ratios, which depend on the materials. The distribution of N atom species was calculated from the deconvolution of the high-resolution spectral regions N 1*s*.

X-ray photoelectron spectroscopy (XPS) was used to determine the elemental composition of the surface of the CTF and the **CTF**-**CNO** materials (Fig. 3A–C, Supplementary Figs. 4 and 5, and Supplementary Tables S1–S3). The distribution of N atom species was calculated from the deconvolution of the high-resolution spectral regions for N 1*s*. All N 1*s* spectra can be deconvoluted into five peaks. The following peaks are typical for covalent triazine skeletons: 398.6 ± 0.4 (triazine N, **N**_**A**_), 399.9 ± 0.3 (pyrrolic N, **N**_**B**_)^35^, 401.5 ± 0.4 eV (tertiary amine in a graphitic network, **N**_**D**_)^31,36^ and 402.7 ± 0.4 eV (quaternary graphitic N, **N**_**E**_)^37^. In our studies, we also observed an additional type of N atom present in the triazine framework at 400.7 ± 0.4 eV, which was attributed to pyrrolo-pyrrolic N (**N**_**C**_)^38^. In the case of pristine CTFs, the ratio of the amount of triazine N (**N**_**A**_) to pyrrolo-pyrrolic N (**N**_**C**_) increased with increasing pyrolysis temperature, from 0.7 (**2CNPP-CTF-500**) to 0.9 (**2CNPP-CTF-700**) (Fig. 3D). A different effect was observed for **2CNPP-CTF-CNOs**, in which the ratio of **N**_**A**_–**N**_**C**_ decreased with increasing temperature. In the case of **2CNPP-CTF-CNO-600**, the ratio of graphitic N (**N**_**D**_) to triazine N (**N**_**A**_) is similar to that of CTFs without the addition of CNOs. However, there is a significant increase in the amount of graphitic N in the hybrid material obtained at 700 °C. Moreover, higher pyrolysis temperatures affect the ordering of the structure and result in the formation of regular CTF.

**Figure 4:**
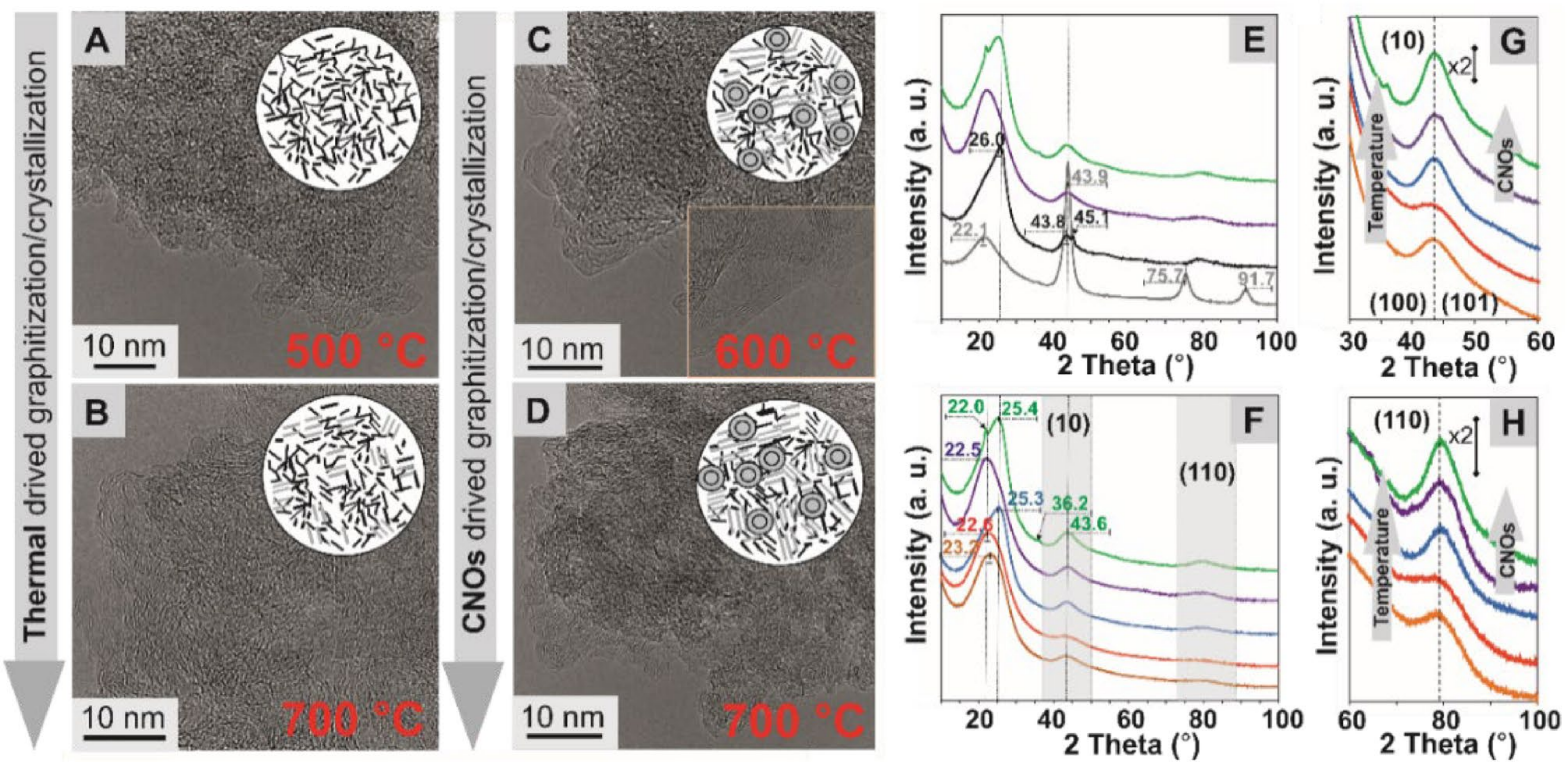
HRTEM images of (**A**) **2CNPP-CTF-500**, (**B**) **2CNPP-CTF-700**, (**C**) **2CNPP-CTF-CNO-600**, and (**D**) **2CNPP-CTF-CNO-700**. XRD patterns (**E**–**H**) of all studied materials: ND (grey line), CNO (black line), **2CNPP-CTF-500** (orange line), **2CNPP-CTF-600** (red line), **2CNPP-CTF-700** (blue line), **2CNPP-CTF-CNO-600** (violet line), and **2CNPP-CTF-CNO-700** (green line). (**G**, **H**) XRD patterns showing the (*hkl*) reflections (10) and (110), which are evidence of the 3D crystalline order of the hybrid materials triggered at the high temperatures of pyrolysis and using spherical nanoplatforms (CNOs).

High-resolution transmission electron microscopic (HRTEM) studies were performed to analyze the influence of the pyrolysis temperature on the structural organization of the CTFs, as well as the effect of adding a functionalized nanostructured spherical platform (CNOs) to the triazine frameworks. The evolution of the structure can be divided into the following stages: the chemical organization of the triazine layers (amorphous phase), formation of a disordered framework, and further graphitization. Amorphous phases and crystalline zones are present in all synthesized materials (Fig. 4A–D and Supplementary Figs. 6 and 7). This structural heterogeneity may affect the higher structural stability and higher conductivity of the carbon materials^39^. In the case of the **CTF-CNO** hybrids, the HRTEM images clearly show the presence of the CNOs in the structures; in addition, a beneficial increase in crystallinity was observed for the materials containing the CNOs compared to the pristine CTFs. We also observed that **2CNPP-CTF-CNO-700** (Fig. 4D) exhibits higher crystallinity than that of the other materials.

The X-ray diffraction (XRD) investigations revealed that the graphitization of CTFs as a function of annealing temperature and the presence of CNOs in the materials occurred (Fig. 4E–H). For the CNOs, the two strongest bands were observed (Fig. 4E,F); the graphite-like peak at 2θ = 26.0°, called the G band, which is attributed to the (002) and (100) planes of graphite; and the D band at approximately 2θ = 43.8°, which corresponds to the (101) basal plane diffraction of the diamond structure (Fig. 4E)^40,41^. The CNO, **2CNPP-CTF**, and **2CNPP-CTF-CNO** profiles show the presence of a mixture of various phases. A broad asymmetric peak in the range between 22 and 27° was observed for all patterns. This peak suggests that some *sp*^2^-bonded carbons are present in all materials. Additionally, due to its asymmetry, the peak can be divided into two subpeaks attributed to two separate forms of carbon, turbostratic carbon (amorphous) and graphene carbon (graphitic carbon)^42^. Increasing the pyrolysis temperature affects the structural organization of the materials. As the width of the (002) peaks decreases, their height increases, and they shift to higher angle values, which indicates a conversion from the amorphous phase to a more graphitized form. Moreover, the (002) reflection is sharper for **2CNPP-CTF-CNO-700**, suggesting that the CNOs also force the formation of microcrystalline structures. Additionally, the (*hk*) and (*hkl*) reflections, such as (10) and (110) (Fig. 4G,H), which are evidence of the 3D crystalline order (at 2θ = 43.8° and 78°)^43^, were also detected for all materials. The separation of the (10) reflection for two (100) and (101) at 2θ = 43.8° and 45.1° for the CNOs, respectively, indicates that this nanostructure exhibits a high level of graphitization^44^. The most ordered structure was observed for **2CNPP-CTF-CNO-700**, and the structural parameters allow its classification as semigraphite (semicrystal material).

### Confirmation of specific pore diameter formation

To confirm the porous nature of the pyrolyzed materials, low-temperature N_2_ adsorption–desorption isotherms were measured. A typical Brunauer–Emmett–Teller (BET) model^45^, in which the procedure proposed by Rouqueol et al. was utilized^46^, and nonlocal density functional theory slit-like carbon pores were applied^47,48^. Low-temperature N_2_ adsorption–desorption isotherms were performed to confirm the porous nature of the pyrolyzed materials. The samples were thermally desorbed at 200 °C in a vacuum for at least 24 h. To obtain the details of the structural changes, the pores were divided into width ranges (up to 1, 1–2, 2–8 and above 8 nm, Table 1).

**Table 1:** Structural parameters of the materials studied, as calculated from the N_2_ adsorption–desorption measurements.

Material	*S*_*BET*_^a^ (m^2^ g^−1^)	*S*_*ext*_^b^(m^2^ g^−1^)	*V*_*tot*_ (cm^3^ g^−1^)	*V*_*micro*_ (cm^3^ g^−1^)	*V*_*1micro*_ (cm^3^ g^−1^)	*V*_*2micro*_ (cm^3^ g^−1^)	*V*_*meso*_ (cm^3^ g^−1^)	*V*_*1meso*_ (cm^3^ g^−1^)	*V*_*2meso*_ (cm^3^ g^−1^)
**2CNPP-CTF-500**	1371	1105	1.124	0.508	0.228	0.280	0.616	0.362	0.254
**2CNPP-CTF-600**	1527	1262	0.889	0.612	0.278	0.334	0.277	0.270	0.007
**2CNPP-CTF-700**	1052	822	1.315	0.318	0.204	0.114	0.997	0.559	0.428
**2CNPP-CTF-CNO-600**	1054	931	0.837	0.359	0.157	0.202	0.478	0.459	0.019
**2CNPP-CTF-CNO-700**	2694	2112	1.944	1.022	0.586	0.436	0.922	0.060	0.862

^a^BET surface area calculated by the method of Rouquerol et al.^46^.

^b^External surface area calculated by the t-plot method with the KJS correction for the layer thickness^49^. The volume of pores (V) was calculated using the DFT model.

*V*_*1micro*_ and *V*_*2micro*_—micropore cumulative volumes from the DFT approach calculated for micropores with widths smaller than 1 nm and in the range of 1–2 nm; *V*_*1meso*_ and *V*_*2meso*_—mesopore cumulative volumes from the DFT approach calculated for mesopores with widths in the range of 2–8 nm and widths larger than 8 nm.

**Figure 5:**
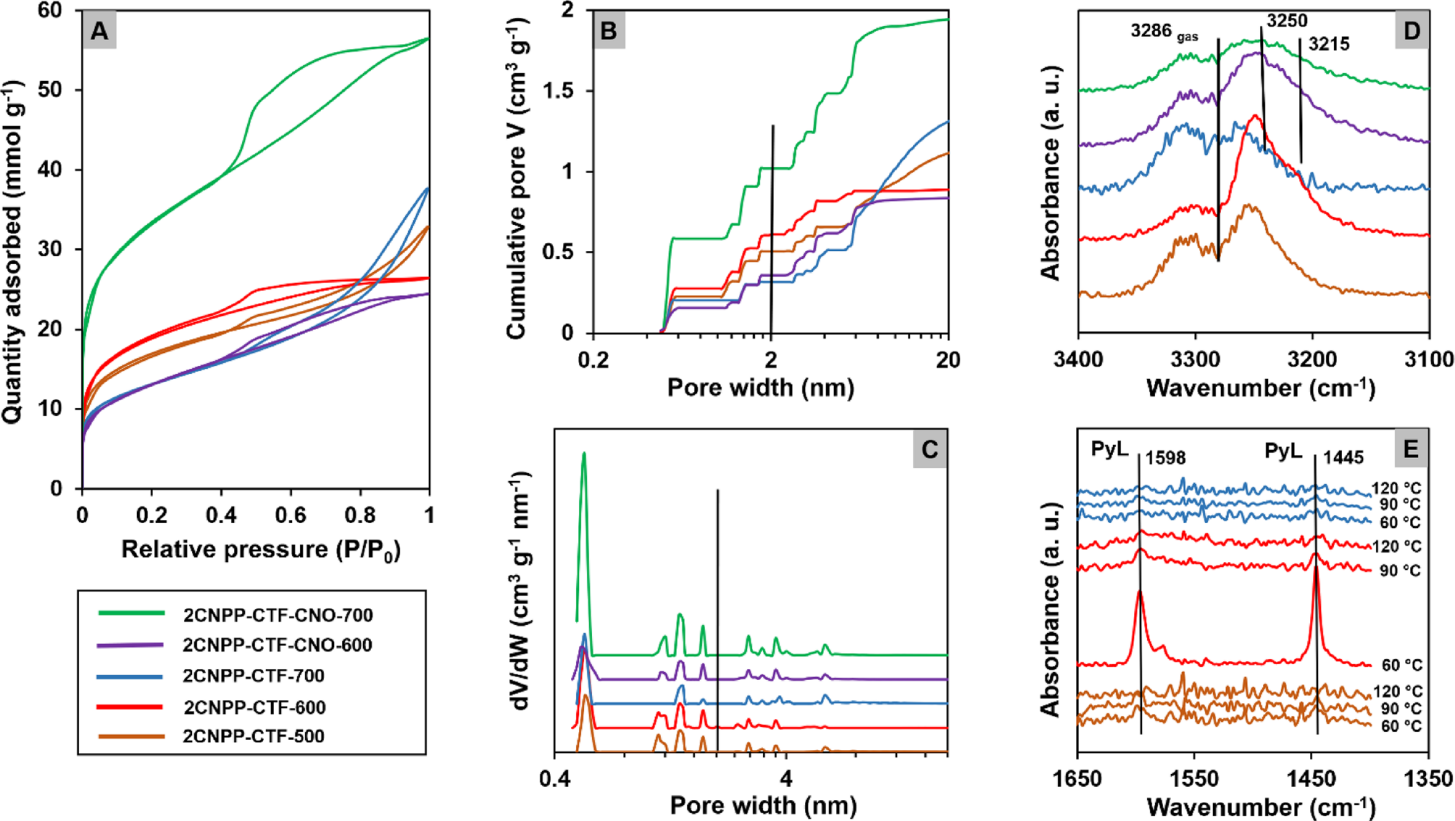
(**A**) N_2_ adsorption–desorption isotherms at 77 K. (**B**) Cumulative and (**C**) differential PSD of **2CNPP-CTF** and **2CNPP-CTF-CNO** materials (the blue vertical line shows the border between the micro- and mesopore sizes). The probe molecule adsorption FT-IR spectra of (**D**) C–H stretching modes of acetylene interacting at − 70 °C with the **2CNPP** and the **2CNPP-CTF-CNO** materials and (**E**) pyridine adsorbed on the studied materials and subsequently desorbed at increasing temperature.

Generally, the profiles of the recorded curves confirmed that all studied materials exhibit a micro-mesoporous character (Fig. 5A). Considering the influence of pyrolysis temperature on **2CNPP-CTF**, we observe an increase in the surface area (*S*_*BET*_) values from 1371 m^2^ g^−1^ for **2CNPP-CTF-500** to 1527 m^2^ g^−1^ for **2CNPP-CTF-600** (Table 1). However, a further increase in the pyrolysis temperature caused the *S*_*BET*_ value to decrease. The increase in the pyrolysis temperature of **2CNPP-CTF** led to a decrease in the volume of micropores (*V*_*micro*_), especially for larger micropores (Fig. 5B,C). Considering the influence of CNO addition to the material (**2CNPP-CTF-CNO-600**), we observed a decrease in the *S*_*BET*_ and the external surface area values. However, the total pore volume was almost preserved compared to that of **2CNPP-CTF-600**. In this case, the use of CNOs reduced the *V*_*micro*_ value. However, the volume of mesopores (*V*_*meso*_), especially in the diameter range of 2–8 nm, was increased. A different tendency was observed for the material that was pyrolyzed at the highest temperature studied (**2CNPP-CTF-CNO-700**). A drastic increase of the *S*_*BET*_ value by approximately 156% (from 1052 for **2CNPP-CTF-700** to 2694 m^2^ g^−1^ for **2CNPP-CTF-CNO-700**) and the *V*_*micro*_ value for the whole diameter range (from 0.318 to 1.022 cm^3^ g^−1^) was observed. Simultaneously, the *V*_*meso*_ value decreased slightly. We can conclude that the highest porosity is kept for the most ordered and graphitized structure of **2CNPP-CTF-CNO-700** (please see XRD data, Fig. 4). The spherical CNOs induce the hierarchical organization of pores with high meso- and microporosity, constituting a permanent skeleton of the structure. Therefore, a significant influence of CNO on the textural and structural properties of the synthesized material is observed. Additionally, the creation of highly porous structures of triazine accompanies its graphitization. This phenomenon is only observed at higher temperatures, in our case, at 700 °C. Therefore, a synergistic effect is observed at 700 °C, the addition of CNO, which organizes the structure in three dimensions, and the simultaneous graphitization of the material. At lower pyrolysis temperatures, the presence of CNOs in the structure prevents a collapse of the microporosity. Thus the addition of CNOs during the organization of CTFs at 700 °C dramatically changed the nanotextural and structural properties of the material, and this change was mainly caused by the development of microporosity (Fig. 5B,C).

### In situ probe molecule adsorption FT-IR studies

The description of Lewis basicity was achieved by acetylene sorption FT-IR studies^50,51^. Binding C_2_H_2_ on the carbon materials (Fig. 5D) results in the appearance of bands at 3253, 3245, and 3223 cm^−1^, which are downshifted compared to that of gaseous acetylene (ν(C–H) at 3286 cm^−1^) due to the formation of weak hydrogen bonds between the probe and basic N atoms in the triazine units, H–C=C–H⋯N. For **2CNPP-CTF-600**, the downshifted component is distinctly asymmetric, and its shape was obtained due to the higher number of adsorption N-sites with different basicity characteristics compared to that of **2CNPP-CTF-500**, for which the spectrum is dominated by the acetylene gaseous phase and the band at 3253 cm^−1^ is slightly overlapped. After annealing at 700 °C, the basicity declines, and the spectrum of **2CNPP-CTF-700** does not exhibit the 3253 cm^−1^ band. For the **2CNPP-CTF-CNO** materials, a new band at 3215 cm^−1^ appears, indicating that additional N-sites appear with a basicity that is higher than that of the **2CNPP-CTF** materials.

The contribution of the 3215 cm^−1^ band to the spectrum, and thus the number of N-sites of high basicity, is lower with an increasing C-to-N ratio. The surface of modified carbon materials can also exhibit weak Brønsted acidity, which is associated with the NH functionality and Lewis acid sites due to the *sp*^3^-hybridized carbon atoms that are created during pyrolysis, for example. The spin density and charge distribution on C atoms can also be altered by neighboring N dopants, resulting in a higher number of defects and thus significantly more active sites^52^. The PyL adduct (1445 cm^−1^) band in the spectrum collected at 60 °C was notably intense for **2CNPP-CTF-600**, while a low number of acidic Lewis sites was found for **2CNPP-CTF-500** and **2CNPP-CTF-700** (Fig. 5E). Pyrolysis at 600 °C results in a significant level of Lewis acidity in addition to the basicity that was detected with C_2_H_2_. None of the CNO-containing materials could bind pyridine, most likely due to the higher amount of CNO-carbon in relation to CTF-carbon. Therefore, compared to CNO structures, the triazine framework should be more susceptible to the formation of defects, e.g., *sp*^3^-hybridized carbon atoms. The acid sites, which are provided by the release of CTF pyridine molecules at a temperature of 120 °C, exhibit a lower acidic strength than that of typical acid sites in oxide materials^53^.

### The energy storage properties of materials

**Figure 6:**
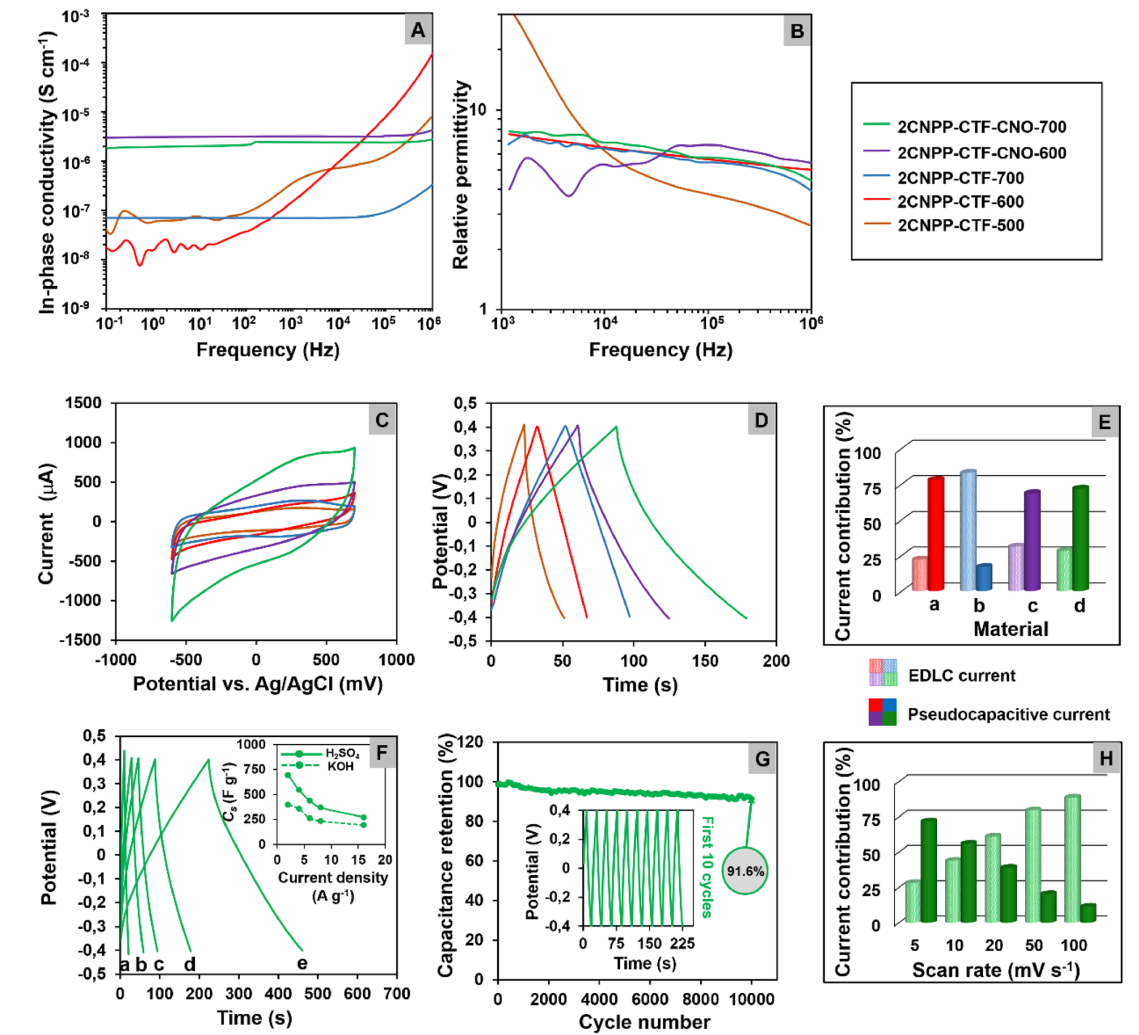
(**A**, **B**) Dielectric spectra of the powder samples at 294 K. (**A**) In-phase component of complex conductivity *ε*′(*f*). (**B**) Real part of the (relative) complex permittivity *ε*′(*f*). Accordingly, the *dc* conductivity *σ*_*dc*_ and dielectric constant *ε*_*S*_ are determined. (**C**) CVs of GCE modified with **2CNPP-CTF** and **2CNPP-CTF-CNO** materials recorded in 1 M H_2_SO_4_ solution at a scan rate of 50 mV s^−1^. (**D**) GCD curves of different materials recorded in 1 M H_2_SO_4_ at a current density of 4 A g^−1^. (**E**) Current contribution for (**a**) **2CNPP-CTF-600**; (**b**) **2CNPP-CTF-700**; (**c**) **2CNPP-CTF-CNO-600**; (**d**) **2CNPP-CTF-CNO-700** in 1 M H_2_SO_4_ at 5 mV s^−1^. (**F**) GCD curves of **2CNPP-CTF-CNO-700** recorded in 1 M H_2_SO_4_ at different current densities [(**a**) 16 A g^−1^; (**b**) 8 A g^−1^; (**c**) 6 A g^−1^; (**d**) 4 A g^−1^; (**e**) 2 A g^−1^]. Inset: Specific capacitance as a function of current density in different electrolytes. (**G**) Capacitance retention as a function of cycle number for **2CNPP-CTF-CNO-700** in 1 M H_2_SO_4_ at a current density of 16 A g^−1^. Inset: First 10 galvanostatic charge/discharge cycles recorded for **2CNPP-CTF-CNO-700**. (**H**) Current contribution for **2CNPP-CTF-CNO-700** at different scan rates in 1 M H_2_SO_4_.

When operated over a broad frequency (*f*) range, broadband dielectric spectroscopy (BDS) can determine the direct current (*dc*) conductance of and capacitance effects by minimizing the undesirable effects of electrode polarization. The real parts of the complex conductivity of the powder samples are as follows (Fig. 6A):*σ*′(*f*) of powder samples follows: *σ*′(*f*) = *σ*_*dc*_ + *Af*^*n*^, where *σ*_*dc*_ denotes the dc conductivity, *A* is a fitting parameter, and *n* is a fractional exponent. The presence of CNOs in materials results in a significant increase in the *dc* conductivity, which is enhanced by approximately two orders of magnitude. The plateau in the *ε*′(*f*) spectra (Fig. 6B) provides an estimate for the (relative) static dielectric constant *ε*_*S*_. The *ε*_*S*_ values of **2CNPP-CTF-CNO-600** and **2CNPP-CTF-CNO-700** are higher than those of **2CNPP-CTF-600** and **2CNPP-CTF-700**, respectively. CNOs have dual roles, which involve enforcing macroscopic charge transport in CTFs and providing large-area carbon multi-layers as possible charge traps. We stress that for BDS on powder samples, trace capacitance is induced by inherent charge carriers, which are trapped due to the heterogeneous structure. Extrinsic ions with various masses and net charges and the different inter-atomic interactions with the atoms that form the internal surface of porous solids can boost the effective dielectric constant, as observed for electrical double layer (EDL) formation in wetted nano- or mesoporous dielectrics, for example. The primary energy mechanism in SCs arises from a reversible electrostatic accumulation of ions on the surface of porous materials, forming an EDL at the electrode surface^54^.

The energy storage properties of the hybrid **2CNPP-CNO**-based CTFs were studied in aqueous solutions using electrochemical methods (Supplementary Figs. 8–11). Cyclic voltammetry (CV) was performed at scan rates from 5 to 100 mV s^−1^. The CV curves of all materials are quasi-rectangular in shape and there are some deviations at approximately 300 mV versus Ag/AgCl (1 M H_2_SO_4_), suggesting that the pseudocapacitive current provides a contribution (Fig. 6C). The galvanostatic charge–discharge (GCD) measurements that were performed in various electrolytes exhibited significant differences in the shape of the recorded curves. The charge/discharge profile that was observed on the GCD curves recorded in 1 M KOH indicated the high resistance of these processes caused by the presence of hydroxide ions (Supplementary Fig. 8). However, the GCD curves recorded in 1 M H_2_SO_4_ revealed a symmetrical, triangular charge/discharge profile, demonstrating the high reversibility of the tested system and the capacitive nature of the material used (Fig. 6D). The slight deviations in the GCD curves from the ideal triangle shape were caused by the pseudocapacitive nature of the tested materials.

The specific capacitances (*C*_*S*_ ) of the synthesized materials included two factors that influenced the electrochemical activity of the material, namely, EDLC and pseudocapacitance. The results of the calculations regarding the contributions of capacitive and pseudocapacitive currents to the total current for the **2CNPP-CTF** and **2CNPP-CTF-CNO** materials at different scan rates are shown in Supplementary Table 4. The analysis of these results confirmed that the share of pseudocapacitive current that was calculated for **2CNPP-CTF-CNO** materials results mainly from the earlier modification of the CNO surface with nitrile-functionalized aryl azide (4-azidobenzonitrile). The contribution of the EDL capacitive current to the pseudocapacitive current was estimated for all synthesized materials (Fig. 6E,H, Supplementary Table 4). The sum of both types of capacitances constitutes the total value of *C*_*S*_. The ratio of EDLC to pseudocapacitance is approximately 1:2 for the materials that contain CNOs. Moreover, the results of the electrochemical analysis in acidic media correspond to the nanotextural properties and XPS analysis results, demonstrating that capacitance is proportional to the N content in all materials. The presence of CNOs in the triazine framework increases the total *C*_*S*_ by three times compared to that of the pristine triazine framework, with an increase from 184 F g^−1^ (**2CNPP-CTF-700**) to 638 F g^−1^ (**2CNPP-CTF-CNO-700**). These are remarkably high capacitance values for COF containing triazines (Supplementary Table 5). A significant increase in the *C*_*S*_ values for **2CNPP-CTF-CNO-600** and **2CNPP-CTF-CNO-700** also indicates that the functionalized CNOs have a strong interaction with **2CNPP-CTF**. As the scan rate increases for **2CNPP-CTF-CNO-700**, the ratio of EDLC to pseudocapacitance significantly increases, indicating that the sizes of pores and supporting electrolyte ions are correlated (Supplementary Table 4). The higher the scan rate is, the lower the response from faradaic reactions, revealing that the diffusion has some limitations (Fig. 6H). The GCD method was used to study **2CNPP-CTF-CNO-700**. The GCD measurements at the different current densities exhibited an almost triangular shape profile with some deviations from non-linearity for the lower values (Fig. 6F). This phenomenon confirmed the high microporosity of the analyzed material^4^.

Other authors have also noticed correlations between the microporosity of the synthesized heteroatom-doped materials, the shape of the GCD curves' triangular profile, and the specific capacity calculated on their basis. In the article by Han et al.^55^ modified electrodes simultaneously promote microporous, heteroatom and multilayer graphene with a proton-conducting substrate, which shows excellent potential in micro-energy storage devices. Other microporous carbons with high specific surface area, high micropore volume and high oxygen content exhibit increased specific capacitance and excellent long-term stability as an electrode material for supercapacitors^56^. For conjugated microporous polymers similar to the covalent triazine structures and reported by Weng et al.^57^ the GCD curves were also triangular with a slight bend, suggesting both pseudocapacitance and EDLC properties. Since a high content of micropores in the synthesized materials was confirmed by N_2_ adsorption/desorption analysis, we concluded that microporosity has a crucial role in obtaining the high specific capacitance of **2CNPP-CTF-CNO-700**. Moreover, the highly graphitic nature of CNOs make **CTF-CNO** hybrids suitable for fast charge and discharge applications^58^. At 4 A g^−1^, **2CNPP-CTF-CNO-700** has a capacitance of 547 F g^−1^. These values correspond to a power density of 1730 W kg^−1^ and an energy density of 43 Wh kg^−1^ (Table 2). Apart from capacitance values, another important characteristic is cyclability. A cycling test at 16 A g^−1^ for **2CNPP-CTF-CNO-700** was performed and indicated that the values dropped to ca. 92% of the starting capacitance after 10,000 cycles (Fig. 6G).

**Table 2:** Specific capacitances, energy densities and power densities calculated from the CV and GCD studies.

Material	*C*_*S*_^a^ (from CV) (F g^−1^)	*C*_*S*_^b^ (from GCD) (F g^−1^)	Energy density^c^ (Wh kg^−1^)	Power density^d^ (W kg^−1^)
Electrolyte: 1 M H_2_SO_4_				
** 2CNPP-CTF-500**	116	187 (4 A g^−1^)	11	1525
** 2CNPP-CTF-600**	151	197 (4 A g^−1^)	17	1821
** 2CNPP-CTF-700**	184	255 (4 A g^−1^)	22	1810
** 2CNPP-CTF-CNO-600**	375	375 (4 A g^−1^)	31	1770
** 2CNPP-CTF-CNO-700**	638	547 (4 A g^−1^)	43	1730
Electrolyte: 1 M KOH				
**2CNPP-CTF-CNO-700**	425	358 (4 A g^−1^)	24	1588

^a^Specific capacitance calculated from the integration of *i*_*c*_*-E* voltammogram (from − 400 to + 400 mV).

^b^Specific capacitance calculated from GCD studies for current density 4 A g^−1^ and potential window from − 400 to + 400 mV versus Ag/AgCl.

^c^Specific energy derived from GCD studies.

^d^Specific power derived from GCD studies.

Porous carbon materials containing triazines are an attractive electrode material used in SC due to their high porosity (Supplementary Table 5). Additionally, after the pyrolysis, they show high thermal stability, a high active porous surface due to a high organization of 2D structure. Various precursors were used to synthesizing triazine systems, which were used as electrode material in SCs (Supplementary Table 5). After synthesis, CTF is subjected to pyrolysis. Generally, the higher the pyrolysis temperature increases the *S*_*BET*_ and the *Cs* values. This relationship was noticed for systems synthesized from terephtalonitrile^59^, pyridine-2,6-dicarbonitrile^60^, or tetrafluoroterephthalonitrile^61^. In some cases, high-temperature pyrolysis of CTFs results in highly porous materials with *S*_*BET*_ value of about 3120 m^2^ g^−1^ (3,5-dicyanopyridine) or *S*_*BET*_ value of about 2500 m^2^ g^−1^ (1,4- or 1,3-dicyanobenzene)^62,63^. However, this does not correlate with the high value of the *Cs* for these systems, below 200 F g^−1^. The highest *Cs* value of 628 F g^−1^ was obtained for polyethynylbenzonitrile^64^. There is only one CTF system synthesized in the presence of carbon nanostructures that consists of *m*-phthalodinitrile and graphene oxide. CTF was pyrolyzed at 800 °C, giving a porous material with *S*_*BET*_ value of 1268 m^2^ g^−1^ and *Cs* is 325 F g^−1^^65^.

Our system, **2CNPP-CTF-CNO-700** possesses an extremely high *Cs* value of 638 F g^−1^ in aqueous solutions. It can be attributed to many factors; mainly the high surface area of the material and its high content of meso- and micropores, semi-crystallinity properties, and the graphitic nature of the carbonaceous material. These provide the opportunity to use this material for supercapacitors.

## Conclusions

This study shows that the covalent immobilization of triazine-based structures on spherical carbon nanostructures results in the organization of micro- and mesopores in a 3D architecture. At the molecular level, the forces that mainly influence the triazine structure organization are the surface chemistry of the CNOs and the experimental conditions used during the preparation of the hybrids. This approach guarantees that the homogenous organization of specifically-sized pores take place in the material and, due to the hybrid nature of the material, a significant increase in its active surface area and electrical conductivity occur; therefore, an improvement in the electrochemical properties also occurs. The presence of micropores results from the surface properties of the CNOs. Meso- and macropores arise mainly from triazine-based structures and intergranular pores between the CNOs.

Our strategy, which is based on the trimerization of nitrile-functionalized pyrrolo[3,2-b]pyrrole molecules, ensures the formation of pores with a well-defined diameter and, due to an appropriately selected pyrolysis temperature, a high semi-crystallinity and conductivity. The carbon nanosheets, which were interconnected, open-channeled, and graphitized, that were formed by the CTFs and organized in a 3D manner on the surface of CNO, were also present in the material. The extremely high *C*_*S*_ value for **2CNPP-CTF-CNO-700** (638 F g^−1^) in aqueous solutions can be attributed to many factors; the high surface area of the material and its high content of micropores, the high content of graphitic N,N-sites with basicity and semi-crystallinity property as well as the graphitic nature of the carbonaceous material.

Herein, the highest capacitance value for systems containing CTFs is reported and, it is one of the highest values ever reported. Moreover, the hybrid systems containing CFTs and CNOs were, for the first time, used as electrodes for SCs. Thanks to the high structural organization and reproducibility, and the high thermal, chemical, and electrochemical stability, the **CTF-CNO** systems are a promising material for use in electrochemistry and electrocatalysis.

## Methods

The materials were prepared through pyrolysis in a Carbolite Gero STF 16/180 tube furnace (with 3216 Controller). High-resolution transmission electron microscopy (HRTEM) was performed using a Titan G2 HRTEM microscope that was operated at 300 kV (FEI company) equipped with a field emission (FEG) gun. HRTEM imaging of the sample microstructure was performed in bright field mode using a CCD camera as a detector. ^1^H and ^13^C NMR spectra were recorded on an Agilent VNMRS system that was operated at 500 MHz for ^1^H NMR and 126 MHz for ^13^C NMR. The chemical shifts δ are given in ppm, are in reference to the solvent peak of CDCl_3_, and are defined at δ 7.26 (^1^H NMR) or δ 77.16 (^13^C NMR). The following abbreviations were used for multiplicities: s (singlet), d (doublet), t (triplet), sept (septet).

High-resolution mass spectra were acquired using a MALDISynapt G2-S HDMS (Waters Corporation, Milford, MA, USA) coupled to a Waters TQD mass spectrometer (electrospray ionization mode ESI-tandem quadrupole). Fourier transform infrared spectroscopy (FT-IR) was performed using a Thermo Scientific Nicolet IN10 MX microscope (USA). The spectra were recorded with a KBr pellet using a microscope in transmission mode. The spectra were collected at a resolution of 4 cm^−1^, and 64 scans were averaged to obtain a single spectrum. The room-temperature Raman spectra were taken with a Renishaw inVia confocal spectrometer (United Kingdom). The parameters used for the Raman measurements were as follows: the wavelength of the laser was 514 nm, the power of the laser beam was 2 mW, and the spectral resolution was 2 cm^−1^. The spectra obtained after normalization were analysed using OMNIC spectroscopy software.

Thermogravimetric (TG) analyses were performed using a Mettler Toledo Star TGA/DSC unit. A sample weighing 2–3 mg was placed in an aluminium oxide crucible and heated from 50 to 900 °C. A heating rate of 10 °C min^−1^ and an argon flow rate of 40 mL min^−1^ were used. An empty pan was used as a reference.

X-ray powder diffraction (XRD). The samples were loaded into glass capillaries (Hampton Research, Glass Number 50) with a diameter of 0.5 mm. The X-ray powder diffraction data were measured using CuKα radiation at 298 K on XtalLAB Synergy diffractometer (Rigaku) equipped with the Hybrid Pixel 2-dimensional detector HyPix-6000HE. For all experiments, the sample-to-detector distance was set to 148 mm, and the data were recorded for the 2θ angle ranging from 10° to 100° using the standard phi scan procedure. In all experiments, the exposure time was 60 s.

X-ray photoelectron spectroscopy (XPS) was performed using an ultrahigh vacuum (UHV) chamber (PREVAC) (base pressure below 10–8 mbar) with a nonmonochromatic AlKα (1486.7 eV; 12 kV; 30 mA) radiation source (VG Scienta SAX 100) and monochromator (VG Scienta XM 780). The emitted photoelectrons were detected using a Scienta R4000 hemispherical analyser. For all samples, a low-resolution survey run (0–1350 eV) at a pass energy of 200 eV was performed. The C 1*s*, O 1*s* and N 1*s* high-resolution spectra were recorded at a pass energy of 20 eV at room temperature. The C 1*s*, O 1*s* and N 1*s* spectra were fitted by Gaussian–Lorentzian functions after Shirley background subtraction was performed. The peaks were fitted using CasaXPS software (Casa Software Ltd.). The C KLL spectra (XAES) were taken from XPS. The first derivative XAES spectra were obtained using a 25-point Savitzky–Golay quadratic polynomial differentiation method.

Nitrogen adsorption–desorption isotherms (77 K) were measured using an ASAP 2020 Plus 2.00A (Micromeritics, USA) adsorption apparatus. Before the adsorption–desorption measurements were taken, all samples were subjected to vacuum evacuation at 473 K for more than 2 h.

Broadband dielectric spectroscopy was performed in the frequency range of 10–1 MHz by employing a Solartron 1206 Frequency Response Analyser equipped with a Novocontrol Broadband Dielectric Converter. An alpha (Novocontrol) active cell, in combination with sample cell BDS2214 for powder or liquids and, alternatively, its self-assembled miniatures, was employed. The spectrometer was monitored by WinDeta software. Voltammetric and galvanostatic charge/discharge studies were performed using a PGSTAT 302N potentiostat (Autolab B.V., Metrohm, Utrecht, the Netherlands) with a three-electrode system (glassy carbon electrode (GCE, 10 × 2 mm) as the working electrode, Ag/AgCl as the reference electrode and Pt mesh (0.25 mm) as the counter electrode). The surface of GCE was modified by deposition of 15 μL of synthesized material solution (3 mg mL^−1^ in EtOH with the addition of conductive carbon paint (CP, SPI Supplies, USA)) and solvent evaporation at RT under an Ar atmosphere. All measurements were carried out in 1 M H_2_SO_4_ or KOH electrolyte solution.

## Materials

Reagents: 4-cyanobenzaldehyde (≥ 98%), 4-isopropylaniline (> 99%), iron(III) perchlorate hydrate Fe(ClO_4_)_3_·H_2_O (crystalline), diacetyl (pure) and anhydrous zinc chloride (≥ 98%) were purchased from Sigma–Aldrich, Poland. The solvents glacial acetic acid (99.5–99.9%), methanol (99.8%), THF (99.5%), ethanol (96%) and concentrated hydrochloric acid (HCl) were purchased from Avantor Performance Materials Poland S.A. Toluene (99.8%), and acetonitrile (99.8%), and dichlorobenzene (99%) were purchased from Sigma–Aldrich, Poland. In the electrochemical measurements, aqueous solutions of sulfuric acid (H_2_SO_4_, 95%, Chempur, Poland) and potassium hydroxide (KOH, Chempur, Poland) were used. All reagents and solvents were used as received. Nanodiamond (ND, powder, 97 wt%, Carbodeon μDiamond®Molto, Carbodeon Oy, Finland) with a crystal size of between 4.2 ± 0.5 nm was used for the preparation of CNOs (an annealing treatment under an inert atmosphere and reduced pressure of ultradispersed ND particles)^66^. 4-Azidobenzonitrile was synthesized according to a literature procedure^67^.

### Synthetic procedures

#### *Synthesis of 1,4-bis*(*4-isopropylphenyl*)*-2,5-bis*(*4-cyanophenyl*)*-1,4-dihydropyrrolo[3,2-b]pyrrole *(*2CNPP*)

4-Cyanobenzaldehyde (1048 mg, 8 mmol) and 4-isopropylaniline (1080 mg, 8 mmol) were added to a mixture of glacial acetic acid (8 mL) and toluene (8 mL). The mixture was stirred at 50 °C for 1 h. After this time, Fe(ClO_4_)_3_·H_2_O (6 mol%, 170 mg, 0.48 mmol) was added, followed by diacetyl (344 mg, 4 mmol). The resulting mixture was stirred in an open flask under an air atmosphere at 50 °C for 12 h. The precipitate was filtered off and washed with water and acetonitrile. The product was purified by crystallization from acetonitrile. After drying under a vacuum, a yellow solid was obtained in a yield of 79% (1.72 g). ^1^H NMR (500 MHz, CDCl_3_, δ): 7.50 (d, *J* = 8.4 Hz, 4H), 7.30 (2d, *J* = 8.4 Hz and 8.3 Hz, 4H), 7.29 (d, *J* = 8.3 Hz, 4H), 7.20 (d, *J* = 8.3 Hz, 4H), 6.50 (s, 2H), 2.99 (sept, *J* = 6.9 Hz, 2H), 1.32 (t, *J* = 6.9 Hz, 12H); ^13^C NMR (126 MHz, CDCl_3_, δ): 146.6, 138.1, 135.0, 133.8, 131.8, 127.6, 127.5, 126.7, 119.4, 112.0, 108.4, 94.6, 44.4, 12.6; HRMS (TOF AP +) *m/z*: [M + H]^+^ calcd for C_38_H_33_N_4_, 545.2705; found, 545.2712.

#### *Synthesis of N-*(*4-cyanophenyl*)*aziridine-CNO *(*CNO-CN*)

CNOs (60 mg) were dispersed in dichlorobenzene (50 mL) by ultrasonication for 30 min. Then, 4-azidobenzonitrile (240 mg) in dichlorobenzene (10 mL) was added, and the mixture was heated at 120 °C for 12 h. The reaction mixture was then ultrasonicated for 1 h and centrifuged for 10 min. The black powder collected at the bottom of the tube was washed three times with toluene and three times with methanol and then dried overnight in an oven (120 °C) to produce modified CNOs (70 mg).

#### Synthesis of 2CNPP-CTF

The ground mixture of 1,4-bis(4-isopropylphenyl)-2,5-bis(4-cyanophenyl)-1,4-dihydropyrrolo[3,2-b]pyrrole (**2CNPP**) (200 mg, 0.37 mmol) and dry ZnCl_2_ (1 g, 7.4 mmol, 20 eq) was placed in a quartz test tube under inert conditions, followed by flame-sealing under a vacuum and slowly heating to the desired temperature (500, 600 or 700 °C) in a tube furnace (heating rate of 1 °C per minute and then 48 h at the desired temperature). After cooling, the tube was opened, and the black monolithic material was ground thoroughly and subsequently washed with 1 M HCl for 24 h, with water and THF several times, and then dried at 120 °C overnight. **2CNPP-CTF-500** 97% yield. **2CNPP-CTF-600** 90% yield. **2CNPP-CTF-700** 88% yield.

#### Synthesis of 2CNPP-CTF-CNO

The ground mixture of 1,4-bis(4-(isopropyl)phenyl)-2,5-bis(4-cyanophenyl)-1,4-dihydropyrrolo-[3,2-b]pyrrole (**2CNPP**) (100 mg, 0.18 mmol), modified CNOs (**CNO-CN**, 5 mg) and dry ZnCl_2_ (0.5 g, 3.7 mmol, 20 eq) was placed in a quartz tube under an inert atmosphere. Next, it was flame-sealed (vacuum closed) and heated to the desired temperature (600 or 700 °C) in the tube furnace (heating rate of 1 °C per minute and then 48 h at the desired temperature). After cooling, the tube was opened, and the black monolithic material was ground thoroughly and subsequently washed with 1 M HCl for 24 h, washed with water and THF several times and then dried at 120 °C overnight. **2CNPP-CTF-CNO-600** 92% yield. **2CNPP-CTF-CNO-700** 89% yield.

## Supplementary Information

Supplementary Information.

## Data Availability

All data supporting the findings of this study are presented graphically or in tables in the paper and Supplementary Information. Raw data provided graphically in this study are available as tabulated values from the corresponding author (M.E.P.-B.) upon reasonable request.
