# Supplementary material for: Three-dimensional organization of pyrrolo[3,2-b]pyrrole-based triazine framework using nanostructural spherical carbon: enhancing electrochemical performance of materials for supercapacitors
Source: Sci Rep. 2023 Jul 3;13:10737. doi: 10.1038/s41598-023-37708-7 (PMC10318046; doi:10.1038/s41598-023-37708-7)
Supplement: Supplementary file 1 — Supplementary Information. [file 41598_2023_37708_MOESM1_ESM.pdf]

Supplementary Information

Three-dimensional Organization of Pyrrolo[3,2-b]pyrrole-Based Triazine Framework Using Nanostructural Spherical Carbon: Enhancing Electrochemical Performance of Materials for Supercapacitors

Agnieszka Hryniewicka[a], Joanna Breczko[a,b], Gabriela Siemiaszko[a], Anthony Papathanassiou[c], Kinga Góra-Marek[d], Karolina A. Tarach[d], Krzysztof Brzezinski[e], Anna Ilnicka[f], Artur P. Terzyk[f], Karolina H. Markiewicz[b], Luis Echegoyen[g]* and Marta E. Plonska-Brzezinska[a]*

[a] Department of Organic Chemistry, Faculty of Pharmacy with the Division of Laboratory Medicine, Medical University of Bialystok, Mickiewicza 2A, 15-222 Bialystok, Poland
[b] Faculty of Chemistry, University of Bialystok, Ciolkowskiego 1K, 15-245 Bialystok, Poland
[c] National and Kapodistrian University of Athens, Physics Department, Condensed Matter Physics Section, Panepistimiopolis, GR 15784 Zografos, Athens, Greece
[d] Faculty of Chemistry, Jagiellonian University in Krakow, Gronostajowa Street 2, 30-387 Krakow, Poland
[e] Department of Structural Biology of Prokaryotic Organisms, Institute of Bioorganic Chemistry, Polish Academy of Sciences, Noskowskiego 12/14, 61-074 Poznan, Poland
[f] Faculty of Chemistry, Nicolaus Copernicus University in Torun, Gagarin Street 7, 87-100 Torun, Poland
[g] Department of Chemistry, University of Texas at El Paso, 500 W. University Ave., El Paso, TX 79968 USA

Table of contents

Supplementary Figure 1. ^{1}H NMR spectrum of 1,4-bis(4-isopropylphenyl)-2,5-bis(4-cyanophenyl)-1,4-dihydropyrrolo[3,2-b]pyrrole (2CNPP). 3

Supplementary Figure 2. ^{13}C NMR spectrum of 1,4-bis(4-isopropylphenyl)-2,5-bis(4-cyanophenyl)-1,4-dihydropyrrolo[3,2-b]pyrrole (2CNPP). 3

Supplementary Figure 3. High-resolution mass spectrum of 1,4-bis(4-isopropylphenyl)-2,5-bis(4-cyanophenyl)-1,4-dihydropyrrolo[3,2-b]pyrrole (2CNPP). 4

Supplementary Figure 4. XPS spectra of the C 1s spectral region of the 2CNPP-CTF and 2CNPP-CTF-CNO materials. 4

Supplementary Figure 5. XPS spectra of the N 1s spectral region of the 2CNPP-CTF and 2CNPP-CTF-CNO materials. 5

Supplementary Table 1. Surface elemental composition of selected carbon samples determined by XPS.......... 5

Supplementary Table 2. Chemical state, positions, FWHM, and relative area percentages of the deconvoluted C 1s peaks obtained from the XPS analyses of 2CNPP-CTF samples. 6

Supplementary Table 3. Chemical state, positions, FWHM, and relative area percentages of the deconvoluted N 1s peaks obtained from XPS analyses.......... 7

Supplementary Figure 6. HRTEM images of 2CNPP-CTFs.......... 8

Supplementary Figure 7. HRTEM images of the 2CNPP-CTF-CNO materials. 9

Supplementary Figure 8. (a) GCD curves of 2CNPP-CTF-700 and 2CNPP-CTF-CNO-700 recorded in 1 M KOH at a current density of 4 A g^{-1} and (b) GCD curves of 2CNPP-CTF-CNO-700 recorded in 1 M KOH at different current densities. 10

Supplementary Figure 9. (a) Cyclic voltammograms of the GCE that was modified with 2CNPP-CTF-700 and 2CNPP-CTF-CNO-700, which was recorded in 1 M KOH solution at a scan rate of 50 mV s^{-1}, and (b) cyclic voltammograms of the GCE that was modified with 2CNPP-CTF-CNO-700, which was recorded in 1 M KOH solution at different scan rates (from 5 to 100 mV s^{-1}).......... 10

Supplementary Figure 10. Cyclic voltammograms of GCE modified with 2CNPP-CTF-CNO-700 recorded in 1 M H_2SO_4 solution at different scan rates (from 5 to 100 mV s^{-1}).......... 11

Supplementary Figure 11. Ragone plot of the specific energy and power of 2CNPP-CTF-CNO-700 in different electrolytes.......... 11

Supplementary Figure 12. TG (solid lines) and DTG curves (dotted lines) of the tested materials. A sample weighing 2-3 mg was placed in an aluminum oxide crucible and heated from 50 °C to 900 °C. A heating rate of 10 °C min^{-1} and an air flow rate of 40 mL min^{-1} were used. An empty pan was used as a reference.

Supplementary Table 4. Current contribution ratios calculated for the synthesized materials at different scan rates in 1 M H_2SO_4. 14

Supplementary Table 5. Electrochemical properties of porous carbon materials containing triazines and carbon nanostructures. 15

References 16

^{1}H NMR and ^{13}C NMR SPECTROSCOPY

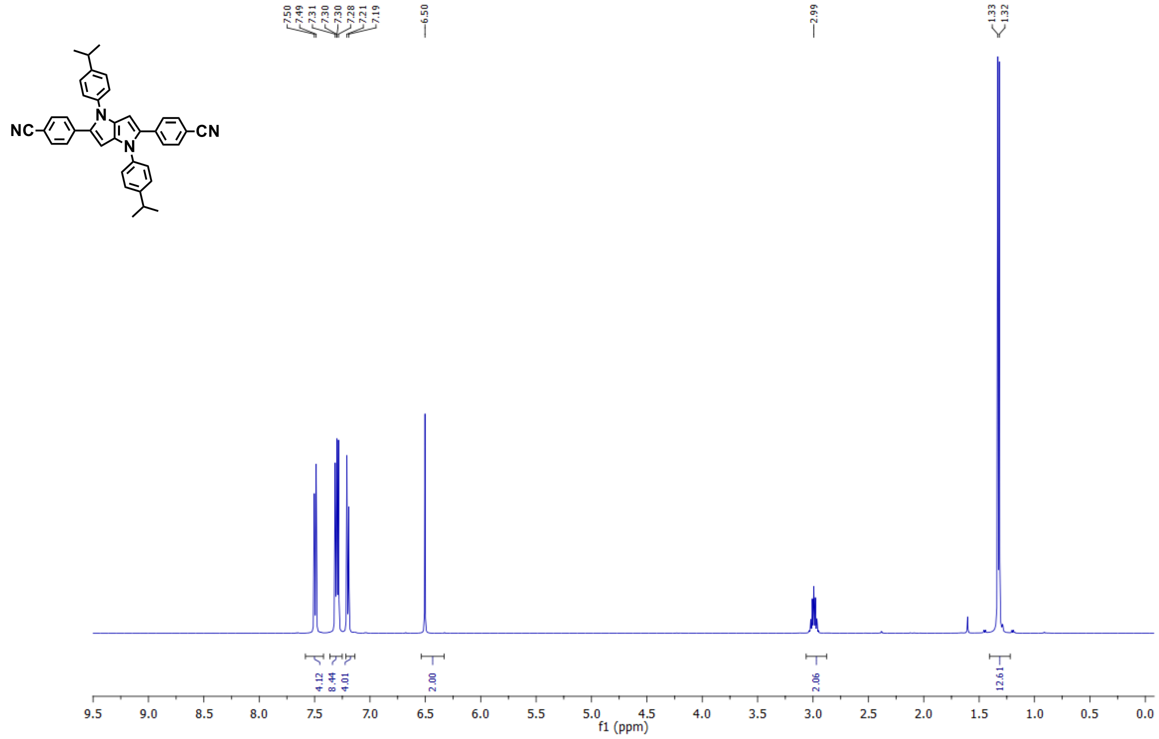

Supplementary Figure 1. ^{1}H NMR spectrum of 1,4-bis(4-isopropylphenyl)-2,5-bis(4-cyanophenyl)-1,4-dihydropyrrolo[3,2-b]pyrrole (**2CNPP**).

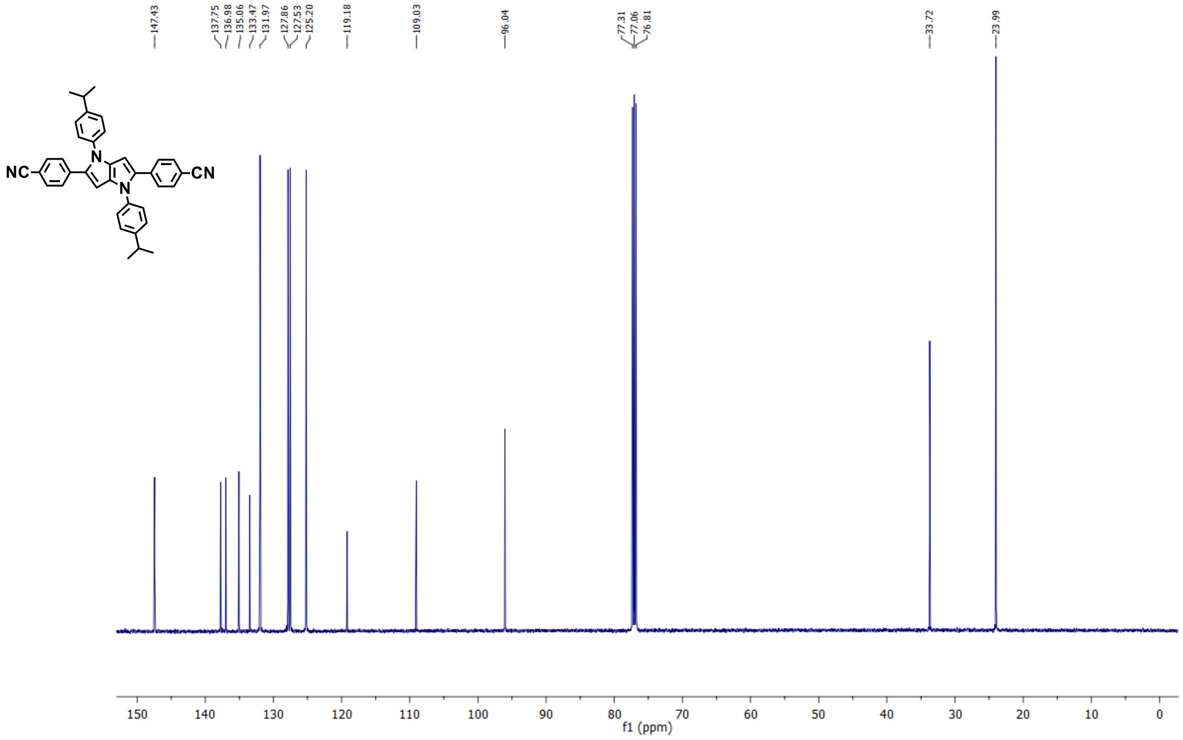

Supplementary Figure 2. ^{13}C NMR spectrum of 1,4-bis(4-isopropylphenyl)-2,5-bis(4-cyanophenyl)-1,4-dihydropyrrolo[3,2-b]pyrrole (**2CNPP**).

MASS SPECTROMETRY

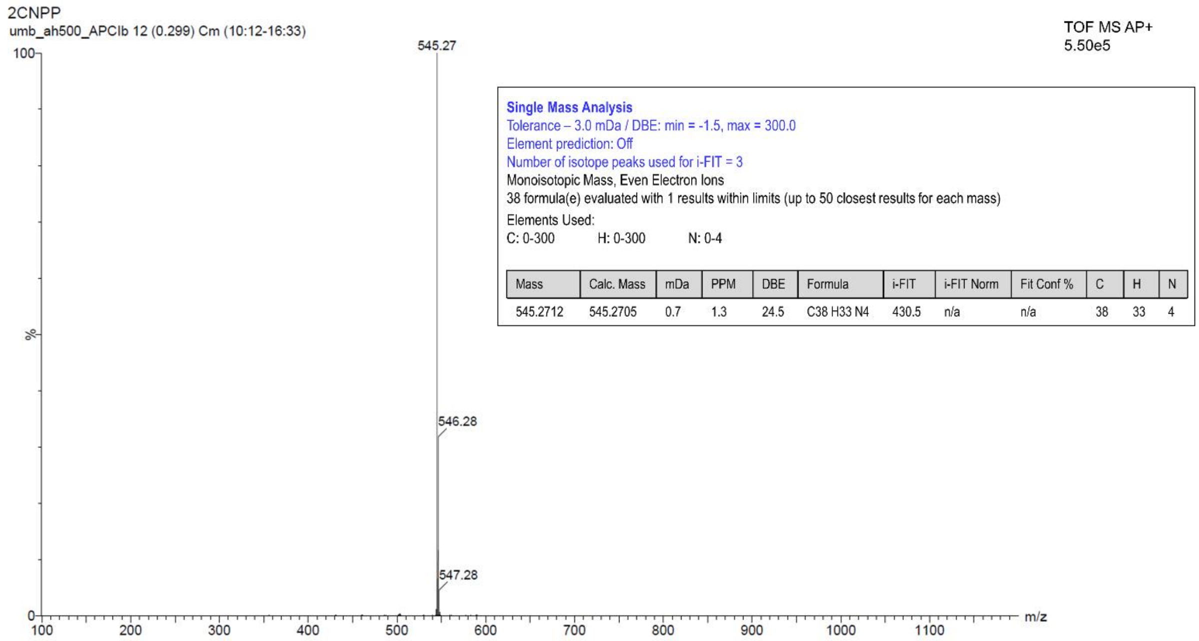

Supplementary Figure 3. High-resolution mass spectrum of 1,4-bis(4-isopropylphenyl)-2,5-bis(4-cyanophenyl)-1,4-dihydropyrrolo[3,2-b]pyrrole (**2CNPP**).

X-RAY PHOTOELECTRON SPECTROSCOPY

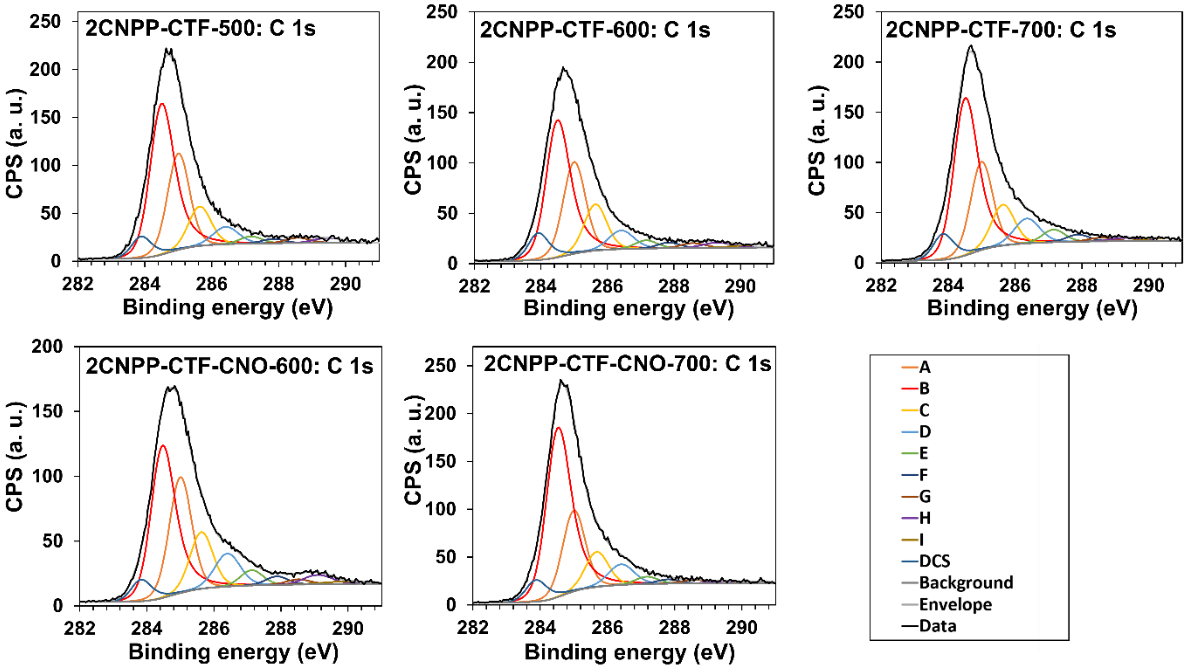

Supplementary Figure 4. XPS spectra of the C 1s spectral region of the **2CNPP-CTF** and **2CNPP-CTF-CNO** materials.

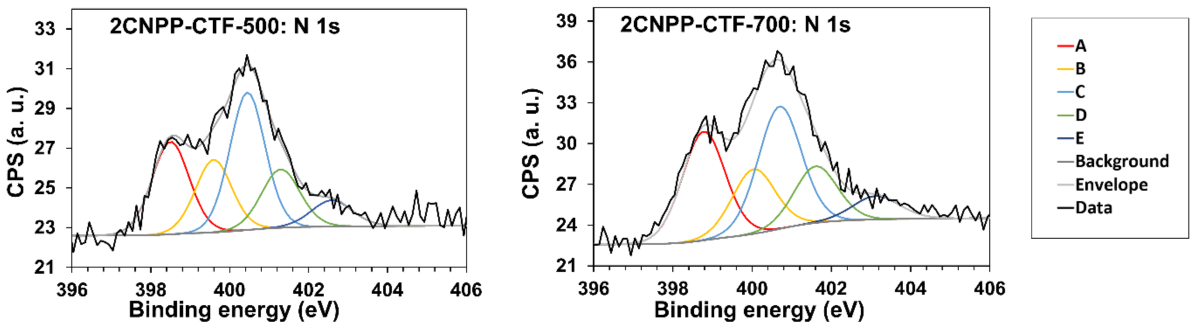

Supplementary Figure 5. XPS spectra of the N 1s spectral region of the **2CNPP-CTF** and **2CNPP-CTF-CNO** materials.

Supplementary Table 1. Surface elemental composition of selected carbon samples determined by XPS.

Sample	Elements (%)				
	C	N	O*	Si*	Al* and/or Cl (trace amounts)
**2CNPP-CTF-500**	91.7	1.6	5.9	0.2	0.5
**2CNPP-CTF-600**	85.5	4.3	8.2	0.5	1.4
**2CNPP-CTF-700**	89.2	6.4	3.6	0.3	0.5
**2CNPP-CTF-CNO-600**	79.0	4.5	11.7	3.5	1.3
**2CNPP-CTF-CNO-700**	91.4	3.6	4.0	0.4	0.5

* The content of these elements may be related to trace glass residues in the pyrolyzed material

Supplementary Table 2. Chemical state, positions, FWHM, and relative area percentages of the deconvoluted C 1s peaks obtained from the XPS analyses of **2CNPP-CTF** samples.

Region	Species	2CNPP-CTF-500			2CNPP-CTF-600			2CNPP-CTF-700			2CNPP-CTF-CNO-600			2CNPP-CTF-CNO-700		
		**Peak (eV)**	**FWHM (eV)**	**% Area**	**Peak (eV)**	**FWHM (eV)**	**% Area**	**Peak (eV)**	**FWHM (eV)**	**% Area**	**Peak (eV)**	**FWHM (eV)**	**% Area**	**Peak (eV)**	**FWHM (eV)**	**% Area**
**C 1s A**	C-H sp^3	285.00	0.77	25.3	285.00	0.78	24.3	285.00	0.77	21.9	285.00	0.78	25.4	285.00	0.78	21.2
**C 1s B**	C=C sp^2	284.46	0.81	46.9	284.46	0.81	42.3	284.47	0.81	46.0	284.43	0.81	38.8	284.49	0.82	52.2
**C 1s C**	C-C sp^3	285.64	0.79	10.6	285.64	0.80	12.5	285.64	0.81	10.6	285.63	0.80	12.7	285.68	0.81	9.4
**C 1s D**	C=N	286.41	0.89	5.3	286.41	0.89	5.6	286.36	0.89	7.1	286.40	0.89	8.2	286.41	0.90	6.2
**C 1s E**	C-O-C	287.16	0.78	1.9	287.16	0.78	2.2	287.15	0.78	3.2	287.12	0.79	3.4	287.17	0.78	1.9
**C 1s F**	C=O	287.84	0.79	1.2	287.84	0.79	1.5	287.88	0.79	1.8	287.88	0.79	1.9	287.81	0.79	1.1
**C 1s G**	C-N	288.57	0.83	1.5	288.57	0.83	1.4	288.57	0.83	1.0	288.51	0.83	1.2	288.56	0.83	1.0
**C 1s H**	O=C-OH	289.48	1.01	1.6	289.31	1.01	1.7	289.31	1.01	1.0	289.10	1.00	2.5	289.50	1.01	0.7
**C 1s I**	π-π*	-	-		290.56	1.64	1.5	290.39	1.14	1.0	289.96	1.69	1.1	290.51	1.00	0.5
**C 1s DCS**	defects in carbon structure	283.85	0.68	5.7	283.88	0.69	7.1	283.82	0.69	6.5	283.80	0.69	4.7	283.84	0.70	5.7

Supplementary Table 3. Chemical state, positions, FWHM, and relative area percentages of the deconvoluted N 1s peaks obtained from XPS analyses.

Region	Species	2CNPP-CTF-500			2CNPP-CTF-600			2CNPP-CTF-700			2CNPP-CTF-CNO-600			2CNPP-CTF-CNO-700		
		**Peak (eV)**	**FWHM (eV)**	**% Area**	**Peak (eV)**	**FWHM (eV)**	**% Area**	**Peak (eV)**	**FWHM (eV)**	**% Area**	**Peak (eV)**	**FWHM (eV)**	**% Area**	**Peak (eV)**	**FWHM (eV)**	**% Area**
**N 1s A**	**triazine** 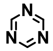	398.50	1.11	24.2	398.64	1.18	28.6	398.78	1.27	28.6	398.22	1.21	25.4	399.02	1.21	11.1
**N 1s B**	**pyrrolic** 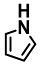	399.59	1.09	18.5	399.73	1.09	18.4	400.04	1.28	17.1	399.46	1.08	25.0	399.80	1.17	19.9
**N 1s C**	**pyrrolo[3,2-b]pyrrolic** 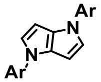	400.45	1.05	34.0	400.49	1.08	31.9	400.69	1.28	32.5	400.34	1.06	27.5	401.07	1.08	22.9
**N 1s D**	**graphitic network** **tertiary amine** 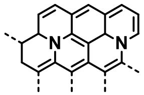	401.29	1.15	15.8	401.35	1.17	15.7	401.60	1.28	15.1	401.10	1.13	18.1	401.94	1.24	33.4
**N 1s E**	**quaternary amine** 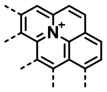	402.58	1.22	7.5	402.91	1.21	5.5	403.12	1.39	6.8	402.41	1.22	4.1	403.04	1.30	12.6

TRANSMISSION ELECTRON MICROSCOPY

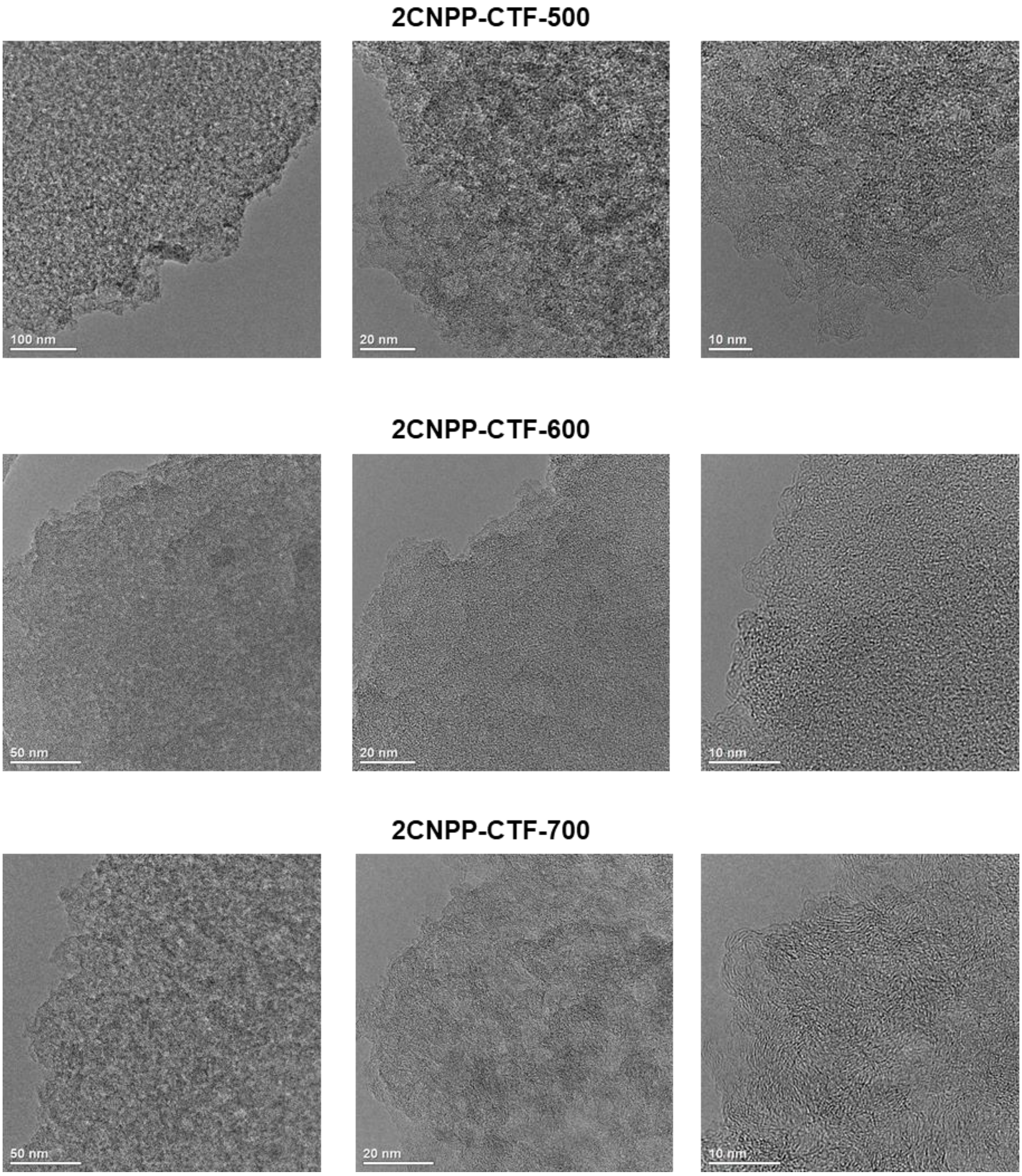

Supplementary Figure 6. HRTEM images of **2CNPP-CTFs**.

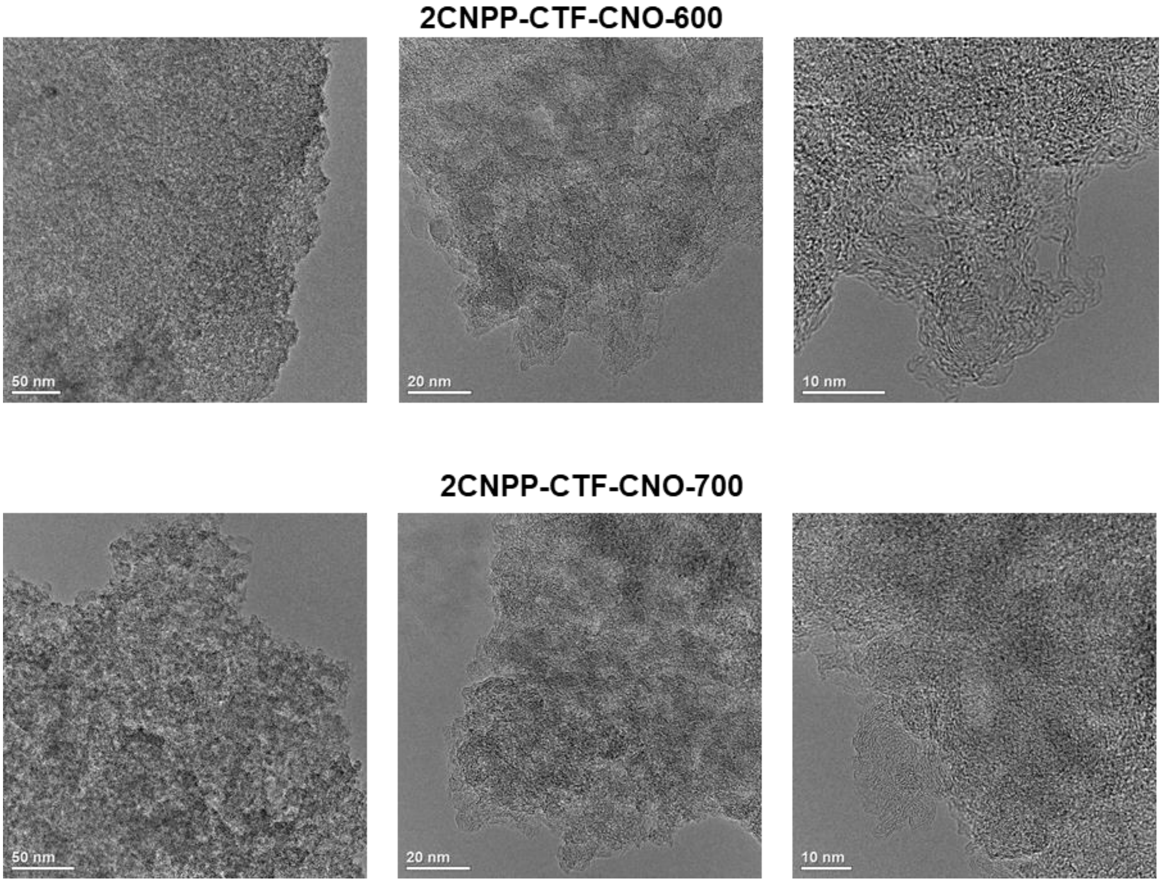

Supplementary Figure 7. HRTEM images of the **2CNPP-CTF-CNO** materials.

GALVANOSTATIC CHARGE/DISCHARGE AND CYCLIC VOLTAMMETRY

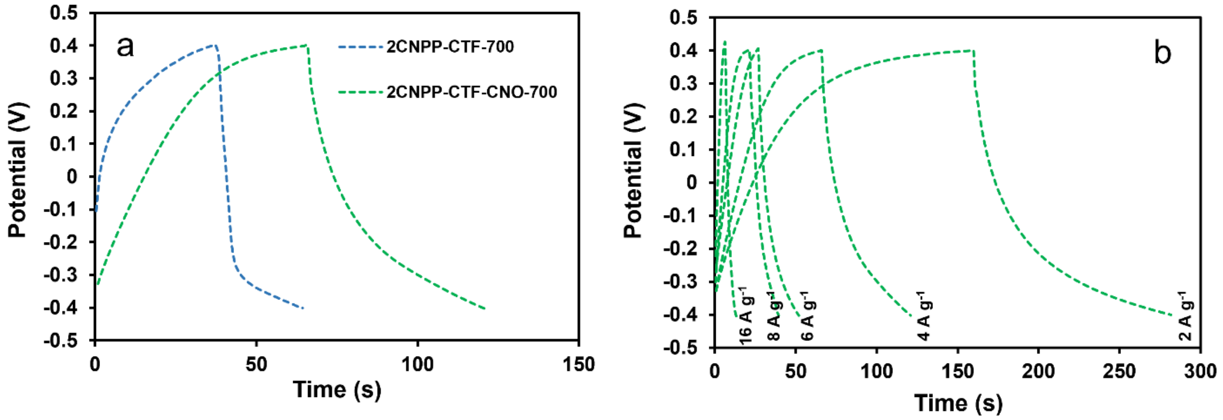

Supplementary Figure 8. (**a**) GCD curves of **2CNPP-CTF-700** and **2CNPP-CTF-CNO-700** recorded in 1 M KOH at a current density of 4 A g^{-1} and (**b**) GCD curves of **2CNPP-CTF-CNO-700** recorded in 1 M KOH at different current densities.

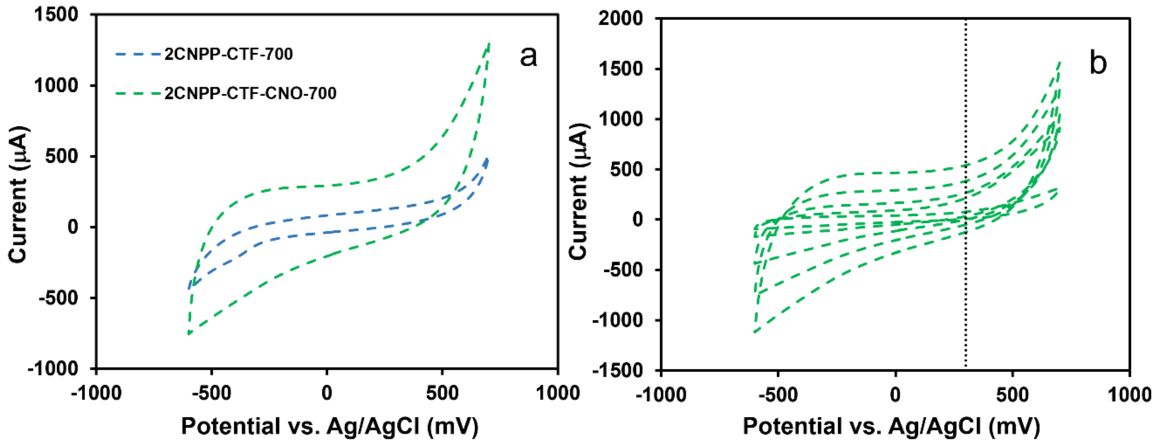

Supplementary Figure 9. (**a**) Cyclic voltammograms of the GCE that was modified with **2CNPP-CTF-700** and **2CNPP-CTF-CNO-700**, which was recorded in 1 M KOH solution at a scan rate of 50 mV s^{-1}, and (**b**) cyclic voltammograms of the GCE that was modified with **2CNPP-CTF-CNO-700**, which was recorded in 1 M KOH solution at different scan rates (from 5 to 100 mV s^{-1}).

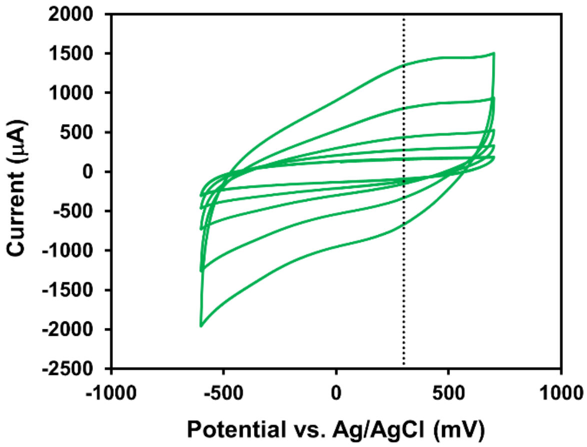

Supplementary Figure 10. Cyclic voltammograms of GCE modified with **2CNPP-CTF-CNO-700** recorded in 1 M H_2SO_4 solution at different scan rates (from 5 to 100 mV s^{-1}).

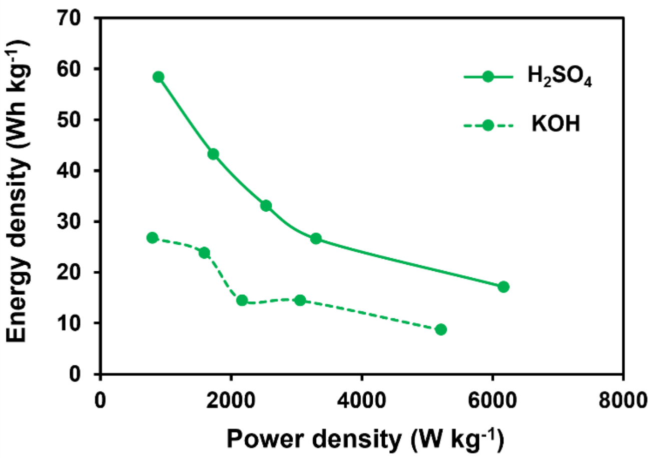

Supplementary Figure 11. Ragone plot of the specific energy and power of **2CNPP-CTF-CNO-700** in different electrolytes.

Based on voltammetric measurements, the specific capacitance (C_s, F g^{-1}) of the materials was determined using CV, and was determined from Equation as follows:

$$C_S = \frac{\int_{E_2}^{E_1} i(E)dE}{2vm(E_1 - E_2)}$$

Where E_1 and E_2 are the initial and final potentials (V), respectively, $\int_{E_2}^{E_1} i(E)dE$ is the integrated current in the potential window, v is the sweep rate (mV s^{-1}) and m is the mass of the active material.

The specific capacitance per single electrode C_s (Fg $^{-1}$) was calculated from the discharge galvanostatic plots at different current densities, following the equation below. For C_s of individual electrodes measured in the three-electrode configuration:

$$C_s = \frac{It_d}{m\Delta V}$$

Where I is the current density, t_d is the discharge time, ΔV is the working voltage once the ohmic drop is subtracted, and m is the mass of the active material in a single electrode.

Energy ($E_{density}$, Wh kg $^{-1}$) and power ($P_{density}$, W kg $^{-1}$) densities were calculated according to the Equations below. The energy and power densities were calculated using the results received from a three-electrode cell to give an energy/power characterization, making a theoretical two-electrode symmetric configuration. We used the appropriate formulas:

$$\boldsymbol{E_{density}} = \frac{\boldsymbol{C_s \cdot (\Delta V)^2}}{\boldsymbol{2 \cdot 3.6}}$$

$$\boldsymbol{P_{density}} = \frac{\boldsymbol{3600 \cdot E_{density}}}{\boldsymbol{t}}$$

THERMOGRAVIMETRIC ANALYSIS

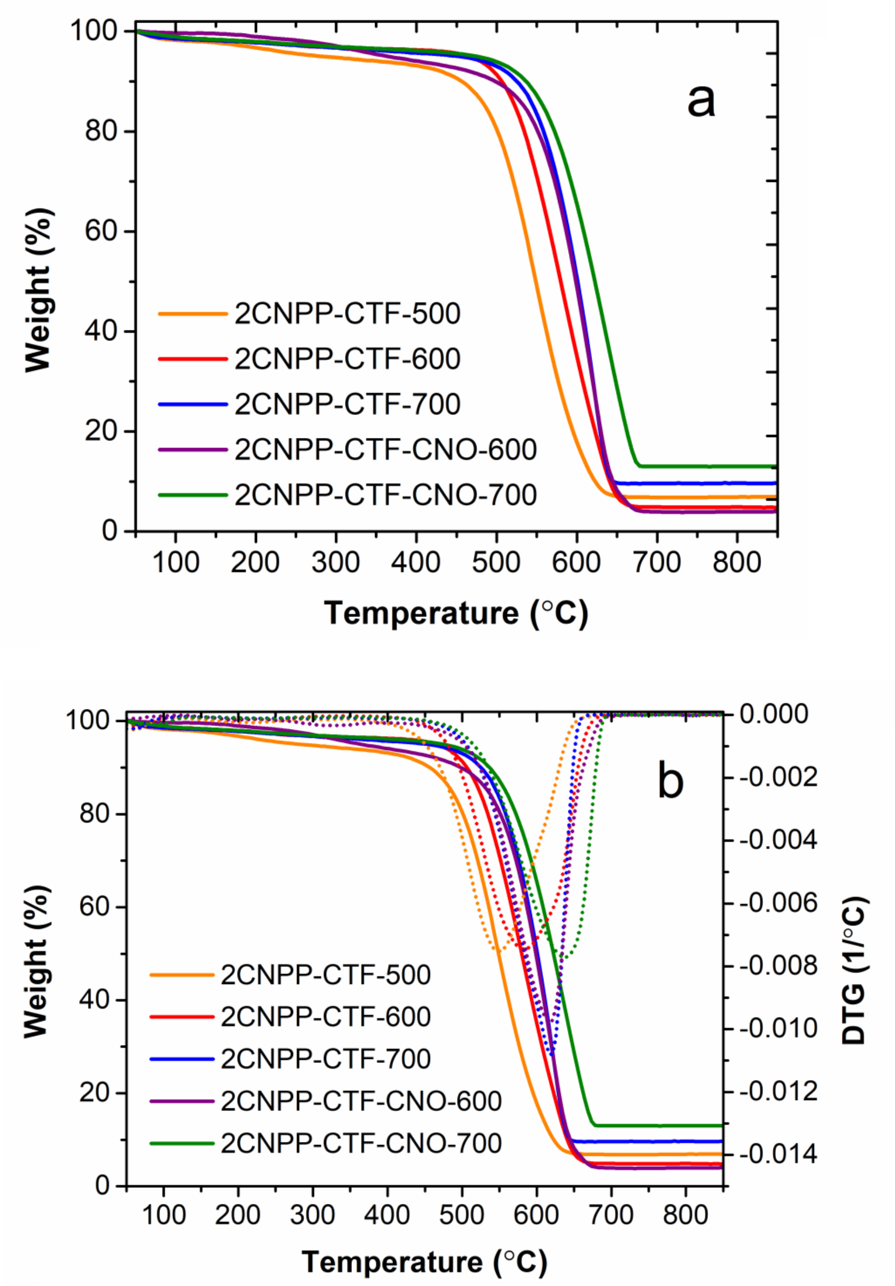

Supplementary Figure 12. TG (solid lines) and DTG curves (dotted lines) of the tested materials. A sample weighing 2-3 mg was placed in an aluminum oxide crucible and heated from 50 °C to 900 °C. A heating rate of 10 °C min^{-1} and an air flow rate of 40 mL min^{-1} were used. An empty pan was used as a reference.

Supplementary Table 4. Current contribution ratios calculated for the synthesized materials at different scan rates in 1 M H_2SO_4.

Material	Electrolyte: 1 M H_2SO_4		
	Scan rate (mV s^{-1})	EDLC current contribution (%)	Pseudocapacitive current contribution (%)
2CNPP-CTF-500	5	66	34
	10	80	20
	20	89	11
	50	95	5
	100	98	2
2CNPP-CTF-600	5	22	78
	10	36	64
	20	53	47
	50	74	26
	100	85	15
2CNPP-CTF-700	5	83	17
	10	91	9
	20	95	5
	50	98	2
	100	99	1
2CNPP-CTF-CNO-600	5	31	69
	10	48	52
	20	65	35
	50	82	18
	100	90	10
2CNPP-CTF-CNO-700	5	28	72
	10	44	56
	20	61	39
	50	80	20
	100	89	11

Supplementary Table 5. Electrochemical properties of porous carbon materials containing triazines and carbon nanostructures.

	Material/Precursor	Temperature of pyrolysis	S_{BET} (m^2 g^{-1})	Content of N (at%)	Capacitance (F g^{-1})[a]	Ref.
**Pristine Triazine**	1,3,5-triphenylbenzene	700-800	711	-	112	1
	TIDN[b]	550; 700	1283; 1527	5.7-8.7	116	2
	3,5-dicyanopyridine	400-700	680-3120	-	141	3
	2,6-diaminanthraquinone	600-800		-	184	4
	1,4-dicyanobenzene<br>1,3-dicyanobenzene<br>2,6-dicyanopyridine	700	1614-2581	-	162-192	5
	4,4'-bipyridine	900	684	5.4	217	6
	fumaronitrile	500-900	485	-	30-220	7
	2,4,6-tris(4-formylphenyl)triazine<br>2,5-diaminohydroquinone dihydrochloride	-	1855	-	256	8
	PYPZ[c]	500-800	981-1464	8.1-3.46	142-256	9
	TIDN[b]	600-900	2003	-	278	10
	terephtalonitrile<br>[1,1'biphenyl]-4,4'-dicarbonitrile<br>[2,2'-bipyridine]-5,5'-dicarbonitrile<br>pyridine-2,6-dicarbonitrile	-	1558<br>1451<br>2278<br>2230	-	220<br>280<br>393<br>324	11
	2,3,5,6-tetrafluoroterephthalonitrile	600-800	1849	15.5-4.4	326	12
	1,4-piperazinedicarboxaldehyde<br>melamine	500-700	748	-	335	13
	2,3,5,6-tetrafluoroterephtalonitrile	400	1345	19.9	379	14
	tetracyanoquinodimethane	400-900	3660	8.13	383	15
	2,4,6-tris(4-aminophenyl)-1,3,5-triazine	600, 700	1057-1237	11.4; 3.0	388	16
	2,6-pyridinedicarbonitrile	800	2795	11.82	406	17
	2,4,6-trichloro-1,3,5-triazine<br>2,6-diaminanthraquinone	800	1993	10.22	468	18
	(5,5',5''-(1,3,5-triazine-2,4,6-triyl)tris(pyridine-2-amine)	-	921-1233	-	546	19
	9,10-dicyanoanthracene	400; 500	400-751	-	589	20
	polyethynylbenzonitrile	800	1954	5.8	628	21
	**1,4-*bis*(4-isopropylphenyl)-2,5-bis(4-cyanophenyl)-1,4-dihydropyrrolo[3,2-b]pyrrole (2CNPP)**	**500-700**	**1052-1572**		**116-184**	**this study**
**Triazine and carbon nanostructures**	*m*-phthalodinitrile<br>graphene oxide	800	1268.5	6.3-9.8	325	22
	**1,4-*bis*(4-isopropylphenyl)-2,5-bis(4-cyanophenyl)-1,4-dihydropyrrolo[3,2-b]pyrrole (2CNPP)<br>CNOs**	**600-700**	**1054-2694**		**375-638**	**this study**

Legend: [a] Determined in aqueous solution in a three-electrode system, [b] TIDN: 4,4'-(1,3,5,7-tetraoxo-5,7-dihydropyrrolo[3,4-f]isoindole-2,6(1H,3H)-diyl)dibenzonitrile, [c] PYPZ: 2,8-bis(4-isocyanophenyl)-2,3,7,8-tetrahydropyridazino[4,5-g]phthalazine-1,4,6,9-tetraone

References

1. Vargheese, S. *et al.* Triazine-Based 2D Covalent Organic Framework-Derived Nitrogen-doped Porous Carbon for Supercapacitor Electrode. *Carbon Lett.* **31**, 879–886 (2021).
2. Hu, F. *et al.* Constructing N, O-Containing Micro/Mesoporous Covalent Triazine-Based Frameworks Toward a Detailed Analysis of the Combined Effect of N, O Heteroatoms on Electrochemical Performance. *Nano Energy* **74**, 104789 (2020).
3. Troschke, E. *et al.* In Situ Generation of Electrolyte inside Pyridine-Based Covalent Triazine Frameworks for Direct Supercapacitor Integration. *ChemSusChem* **13**, 3192–3198 (2020).
4. Luo, B., Chen, Y., Zhang, Y. & Huo, J. Nitrogen-rich Anthraquinone–Triazine Conjugated Microporous Polymer Networks as High-Performance Supercapacitor. *New J. Chem.* **45**, 17278–17286 (2021).
5. Baumann, D., Lee, C., Wan, C., Sun, H. & Duan, X. Hierarchical Porous Carbon Derived from Covalent Triazine Frameworks for High Mass Loading Supercapacitors. *ACS Materials Lett.* **1**, 320–326 (2019).
6. Lei, Z. *et al.* Nitrogen-doped Porous Carbon with Brain-like Structure Derived from Quaternary Bipyridinium-type Framework for Efficient Oxygen Reduction Electrocatalysis and Supercapacitors. *ChemElectroChem* **6**, 848–855 (2019).
7. Wang, D.-G. *et al.* Synthesis and Morphology Evolution of Ultrahigh Content Nitrogen-Doped, Micropore-Dominated Carbon Materials as High-Performance Supercapacitors. *ChemSusChem* **11**, 3932–3940 (2018).
8. El-Mahdy, A. F. M. *et al.* A Hollow Microtubular Triazine- and Benzobisoxazole-Based Covalent Organic Framework Presenting Sponge-Like Shells That Functions as a High-Performance Supercapacitor. *Chem. Asian J.* **14**, 1429–1435 (2019).
9. Cao, R. *et al.* Bottom-up Fabrication of Triazine-Based Frameworks as Metal-Free Materials for Supercapacitors and Oxygen Reduction Reaction. *RSC Adv.* **11**, 8384–8393 (2021).
10. Kim, M. *et al.* High Performance Carbon Supercapacitor Electrodes Derived from a Triazine-Based Covalent Organic Polymer with Regular Porosity. *Electrochim. Acta* **284**, 98–107 (2018).
11. Zhang, Y. *et al.* Rational Design of Covalent Triazine Frameworks Based on Pore Size and Heteroatomic Toward High Performance Supercapacitors. *J. Colloid Interface Sci.* **606**, 1534–1542 (2022).
12. Gao, Y. *et al.* Fluorine/Nitrogen co-Doped Porous Carbons Derived from Covalent Triazine Frameworks for High-Performance Supercapacitors. *ACS Appl. Energy Mater.* **4**, 4519–4529 (2021).
13. Li, L. *et al.* Ultrastable Triazine-Based Covalent Organic Framework with an Interlayer Hydrogen Bonding for Supercapacitor Applications. *ACS Appl. Mater. Interfaces* **11**, 26355–26363 (2019).
14. Gao, Y. *et al.* Halogen-Functionalized Triazine-Based Organic Frameworks Towards High Performance Supercapacitors. *Chem. Eng. J.* **400**, 125967 (2020).
15. Li, Y. *et al.* Conductive Microporous Covalent Triazine-Based Framework for High-Performance Electrochemical Capacitive Energy Storage. *Angew. Chem. Int. Ed.* **57**, 7992–7996 (2018).
16. Deka, N., Patidar, R., Kasthuri, S., Venkatramaiah, N. & Dutta, G. K. Triazine Based Polyimide Framework Derived N-Doped Porous Carbons: a Study of Their Capacitive Behaviour in Aqueous Acidic Electrolyte. *Mater. Chem. Front.* **3**, 680–689 (2019).
17. Wu, C. *et al.* In Situ Nitrogen-Doped Covalent Triazine-Based Multiporous Cross-Linking Framework for High-Performance Energy Storage. *Adv. Electron. Mater.* **6**, 2000253 (2020).
18. Song, Z. *et al.* Self-Assembled Carbon Superstructures Achieving Ultra-Stable and Fast Proton-Coupled Charge Storage Kinetics. *Adv. Mater.* **33**, 2104148 (2021).
19. Haldar, S., Kushwaha, R., Maity, R. & Vaidhyanathan, R. Pyridine-Rich Covalent Organic Frameworks as High-Performance Solid-State Supercapacitors. *ACS Materials Lett.* **1**, 490–497 (2019).
20. Mohamed, M. G. *et al.* Ultrastable Covalent Triazine Organic Framework Based on Anthracene Moiety as Platform for High-Performance Carbon Dioxide Adsorption and Supercapacitors. *Int. J. Mol. Sci.* **23**, 3174 (2022).
21. Mohamed, M. G., EL-Mahdy, A. F. M., Takashi, Y. & Kuo, S.-W. Ultrastable Conductive Microporous Covalent Triazine Frameworks Based on Pyrene Moieties Provide High-Performance CO_2 Uptake and Supercapacitance. *New J. Chem.* **44**, 8241–8253 (2020).
22. Peng, L. *et al.* Nitrogen Doped Carbons Derived From Graphene Aerogel Templated Triazine-Based Conjugated Microporous Polymers for High-Performance Supercapacitors. *Front. Chem.* **7**, 142 (2019).
